# Nutritional Supplementation Benefits in *Caenorhabditis elegans* under Developmental Disruption and Stress Conditions

**DOI:** 10.1021/acsomega.4c10748

**Published:** 2025-07-17

**Authors:** Daiana Silva Ávila, Camila Milagres Macedo Pereira, Iverson Conrado Bezerra, Gabriel Pedroso Viçozzi, Alex Aparecido Rosini Silva, Artur José da Silva, Heloísa Aiolfi Padilha, Emilly de Souza Cordeiro, Aline Castro Silva, Danilo Cardoso de Oliveira, Katarine Gabriely Aurista do Nascimento, Julianne de SantanaCavalcante, Clarice Beatriz Gonçalves Silva, Roberto Afonso da Silva, Matheus Chimelo Bianchini, José Luiz de Lima Filho, Andreia M. Porcari, Priscila Gubert

**Affiliations:** † Graduate Program in Biochemistry, 186060 Federal University of Pampa (UNIPAMPA), Uruguaiana 96460-000, Brazil; ‡ Graduate Program in Biological Sciences, Toxicological Biochemistry, Federal University of Santa Maria, Santa Maria 97105-900, Brazil; § Keizo Asami Institute, iLIKA, 28116 Federal University of Pernambuco, Recife 50670-901, Brazil; ∥ Graduate Program in Biology Applied to Health, PPGBAS, Federal University of Pernambuco, Recife 50670-901, Brazil; ⊥ MS^4^Life Laboratory of Mass Spectrometry, Health Sciences Postgraduate Program, 154623 Universidade São Francisco, Bragança Paulista, São Paulo 12916-900, Brazil; # Graduate Program in Biomedical Science, Federal University of Fronteira Sul (UFFS), Chapecó 89815-899, Brazil; ¶ Graduate Program in Pure and Applied Chemistry, POSQUIPA, Federal University of Western of Bahia, Bahia 41300-770, Brazil

## Abstract

Autism spectrum disorder (ASD) is a developmental and neurological condition that impacts an individual’s behavior, communication, social interaction, and learning abilities. This disease is complex and involves different mechanisms, and therefore, modeling is a major challenge. Some features can be reproduced in different animal models to investigate therapeutic approaches. Here, we proposed a new simple model to induce development delay by using the nematode *Caenorhabditis elegans* and dithiothreitol (DTT) as a chemical agent. In order to investigate a complementary treatment, a commercial supplement and its isolated components (vit. B12, B1, B6, and B9), curcumin, and palmitoylethanolamide (PEA) were used to revert the oxidative stress, development impairments, and metabolomic changes caused by DTT. Furthermore, computational tools predicted pharmacokinetic properties (SwissADME) and possible pathways (enrichment analysis) linked to ASD, an important neurodevelopmental disorder. The supplement and its components (curcumin, PEA, vit. B12, B6, and B9) partially alleviated the delay in larval progression and completely recovered the GABAergic neurodevelopmental impairments (supplement, curcumin, vit. B6, B9, and B12) caused by DDT. Vitamin B9, B12, and supplement partially protected mortality against the oxidative stressor Paraquat. Curcumin, vit. B1, B6, and supplement showed potential protection against coexposure to DTT by reducing DAF-16 migration to the nucleus compared to DTT. Vitamins B1, B9, and B12 coexposure to DTT positively modulated SOD-3 expression. Amino acids, carnitines, and lipids revealed by LC–MS analysis enabled group differentiation and pathway analysis, indicating potential signaling molecules. In silico analysis predicted that these components may interact with pathways linked to ASD pathogenesis such as immunomodulation, synaptic pruning, and complex behavior regulation. Our data indicate that DTT is a good chemical model to induce developmental disorders and that the supplement, with all its components associated, is a promising therapy to be investigated in ASD.

## Graphical abstract

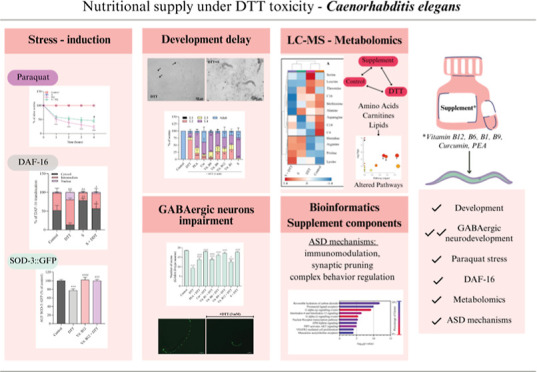

## 1 Introduction

Autism spectrum disorder (ASD) encompasses a group of neurodevelopmental conditions characterized by deficits in social communication alongside restricted and repetitive behaviors or interests. Neurodevelopmental disabilities, including autism, attention-deficit hyperactivity disorder, dyslexia, and other cognitive impairments, affect millions of children worldwide, and some diagnoses seem to be increasing in frequency. Some results provide scientific evidence of the association that exists between the environmental neurotoxins and various neurodevelopmental disorders.^1^ Industrial chemicals that injure the developing brain are among the known causes for this rise in prevalence.^2^ Recent data from the Centers for Disease Control and Prevention’s Autism and Developmental Disabilities Monitoring Network indicate that approximately 1 in 36 children are diagnosed with ASD,^3^ emphasizing the urgent need for scientific effort.

The term “spectrum” refers not only to the wide range of symptoms observed in ASD, varying in severity and progression, but also to the underlying physiological diversity. The significant heterogeneity in both clinical presentation and biological underpinnings makes treatment decisions particularly challenging, as a one-size-fits-all approach fails to address the complexity of the condition. The physiological complexity of ASD is attributed to an interplay of environmental, genetic, and epigenetic factors, including histone modifications and DNA methylation.^4,5^ Disruptions in the homeostasis of neurotransmitter systems, such as gamma-aminobutyric acid (GABA), glutamate, serotonin, dopamine, and *N*-acetyl aspartate, have been linked to ASD, reflecting its multifaceted pathophysiology.^6^

Disrupted folate and transmethylation pathways and other causes of methylation impairment have been linked to oxidative stress on an ASD group and has specific metabolomic markers,^7–9^ which suggest that oxidative stress and methylation defects may not only contribute to ASD development but also serve as biomarkers for defining a metabolic endophenotype within the spectrum. This endophenotype, marked by disrupted redox homeostasis^10^ and impaired metabolic and epigenetic resilience,^11^ represents a potential target for personalized therapeutic interventions, emphasizing the need to address the unique biological mechanisms underlying each ASD subtype.

Despite the advances in understanding the heterogeneity of ASD, there is a significant clinical gap for patients’ classification into specific endophenotypes.^12^ As a result, most interventions remain generalized and insufficient to address symptoms in individuals,^13^ underscoring the urgent need for robust research to stratify ASD based on unique neurobiological and metabolic profiles.

Current drugs used for ASD address behavioral symptoms; however, they are ineffective for the core symptoms (social communication difficulties, restricted interests, and repetitive behaviors).^14^ They also fail to address the individuality of endophenotypes, such as metabolic subtypes characterized by oxidative stress and impaired methylation. Dietary supplements have been proposed as a promising therapeutic avenue for addressing symptoms of ASD,^15^ particularly for subgroups with distinct metabolic vulnerabilities. Among these supplements, B-complex vitamins, such as vit. B1, B6, B9, and B12, are of particular interest due to their role in correcting methylation deficits,^16^ a hallmark of the metabolic endophenotype associated with methylation defects and disturbances on folate pathways.

Studies have shown that antioxidants may play a beneficial role in addressing oxidative stress and related metabolic dysfunctions in ASD.^17^ Among these, Modafferi et al. reviewed the potential of antioxidant-rich mushrooms as treatments for ASD, proposing *C. elegans* as a practical model to investigate the molecular mechanisms involved.^18^ Other natural compounds, such as curcumin, resveratrol, naringenin, sulforaphane,^19^ and palmitoylethanolamide (PEA),^20^ have also demonstrated their potential in reducing oxidative stress and targeting oxidative stress. Additionally, clinical trials have identified a subgroup of ASD patients who respond exceptionally well to supplements,^21–23^ suggesting that some individuals may represent a differential responder group linked to specific endophenotypes.

In order to evaluate these promising molecules, animal models are needed. Multiple animal models of ASD, including transgenic mice models, have been developed to clarify the etiology and prospect new therapeutic strategies.^14^ However, the variability on the spectrum poses challenges for animal modeling: whereas chemical models such as valproic acid (VPA) and polyinosinic/polycytidylic acid (poly I/C)^24^ have provided valuable insights in animal models, they fail to capture the full ASD complexity, particularly endophenotypes associated with other metabolic dysregulations.

Simple experimental models can be used to replicate endophenotype mechanisms and explore new therapies. In this perspective, *C. elegans* can be used due to their conserved neuronal pathways and simplicity in studying some of the neurodevelopmental disorder features that are also observed in ASD patients. Furthermore, worms are becoming a preferred organism model for toxicity testing to comply with the 3R (replace, reduce, and refine) concept, reducing the use of vertebrate animal testing.^25^ Using worms to model neurodevelopmental disorders like ASD is an emerging topic that harbors great, untapped potential.^26^

Here, we propose testing a new chemical model for understanding some aspects of development and ASD in *C. elegans*. We developed a chemical model in *C. elegans* using dithiothreitol (DTT), which can modulate methylations to induce developmental delay as a phenotype. In *C. elegans*, DTT increases the expression of R08E5.3, an *S*-adenosyl-methionine (SAM)-dependent methyltransferase, and modulates methionine–homocysteine cycle activity.^27^ DTT can also cause *C. elegans* proteotoxic stress and reductive stress, which are attributed to ASD etiology.^27,28^ We also tested a new supplement based on B-vitamins, curcumin extract, and PEA on our model.

Considering the promising findings, further research is required to confirm the efficacy and safety of supplements and refine stratification methods for targeted interventions. In this context, our study aimed to evaluate the effects of one supplement based on B-vitamins, curcumin extract, and PEA and these components individually on key factors such as methylation, oxidative stress, regulation of antioxidant response machinery, developmental delays, behavioral impairments, and metabolomic changes induced by DTT as a chemical model for ASD-related features in *C. elegans*. Additionally, in silico analyses were conducted to predict the chemical properties, biological metabolism, and activity of these molecules, with the goal of optimizing the supplement’s pharmacokinetics.

## 2 Materials and Methods

### 2.1 Chemicals

Sodium chloride P.A (Qumica moderna, SP, Brazil), hydrochloric acid P.A A.C.S. (Anidrol, SP, Brazil), sodium biphosphate P.A.C.S (Synth, SP, Brazil), potassium phosphate monobasic anhydrous P.A (Synth, SP, Brazil), sodium hydroxide (Synth, SP, Brazil), and methanol (xodo cientfica, SP, Brazil). NGM medium: agar (EMPROVE, Darmstadt, Germany), peptone (Sigma aldrich, St. Louis, EUA), and sodium chloride P.A. (Quimica moderna, SP, Brazil). M9 buffer: sodium phosphate dibasic P.A A.C.S (Sigma-Aldrich, St. Louis, EUA), potassium phosphate bibasic P.A (Synth, SP, Brazil), and sodium chloride P.A (Quimica moderna, SP, Brazil). Uranyl acetate (Fisher scientific, EUA) and OP50 *Escherichia coli* (Fisher scientific, EUA). Unlabeled amino acid and acylcarnitine standards were purchased from Merck (Merck KGaA, Darmstadt, Germany). Acetonitrile and methanol HPLC–MS-grade solvents were obtained from J.T. Baker. All other reagents were of analytical grade and were obtained from local suppliers.

### 2.2 *C. elegans* Strains and Maintenance

Worms were cultivated in Petri dishes containing a nematode growth medium (NGM, 3 g NaCl, 2.5 g peptone, 17 g agar, and 975 mL of autoclaved H_2_O; further added 1 mL of 1 mol L^–1^ CaCl_2_, 1 mL of 5 mg mL^–1^ cholesterol in ethanol, 1 mL of 1 mol L^–1^ MgSO_4_, and 25 mL 1 mol L^–1^ KPO_4_ buffer)^29^ and fed with *E. coli* OP50 under controlled temperature (20 °C) and humidity (>95%) (Panasonic Healthcare Company of North America, MIR-254-PA). The population was synchronized to obtain all worms at the same larval stage. For this, a lysis solution was prepared (NaOH 1 M, NaClO 1%, and distilled H_2_O) to break the cuticle of pregnant hermaphrodites and release the eggs. Eggs were kept in the M9 buffer (3 g L^–1^ KH_2_PO_4_, 6 g L^–1^ Na_2_HPO_4_, 5 g L^–1^ NaCl, and 1 mM MgSO_4_) until hatching and reaching the first larval stage (L1). All the strains employed were submitted to the same process. The strains used consisted of N2 (wild-type, Bristol), CF1553 muIs84 [(pAD76)*sod-3*p::GFP + *rol-6*(su1006)], TJ356 zIs356 [*daf-16p*::*daf-16*a/b::GFP + *rol-6*(su1006)] IV, and EG1285 oxIs12 [*unc-47p*::GFP + *lin-15*(+)]. The strains of *C. elegans* and *E. coli* were obtained from the Caenorhabditis Genetics Center (CGC, Minnesota, USA).

### 2.3 Exposure Protocol

The supplement (303 mg L^–1^, Endocan) and the isolated components (PEA, Levagen plus, 200 mg L^–1^), curcumin (Hydrocure, 80 mg L^–1^), and B complex vitamins: B1 (1 mg L^–1^), B6 (49 mg L^–1^), B9 (0.3 mg L^–1^), and B12 (0.0048 mg L^–1^), and DTT (3 mM; Sigma-Aldrich; CAS: 3483-12-3) were dissolved in ultrapure water prior to the experiments. PEA and curcumin are difficult to be dissolved in water. However, the chosen supplement contains a formulation that rapidly disperses in water, eliminating the need for toxic solvents for *C. elegans*. Both the supplement and all of the isolated components were dissolved in ultrapure water to prepare a stock solution. The solutions (supplement or its components or DTT) were added to the surface of the NGM plate seeded with *E. coli* OP50 and incubated in a refrigerator for 24 h prior to starting the experiment. Worms at the L1 larval stage were exposed on NGM plates to the supplement or its components, with or without DTT, for 44 h or until 72 h for developmental evaluations. The chosen concentrations were based on previous work and standardized to represent the same proportion of the supplement (Figure 1).

**Figure 1:**
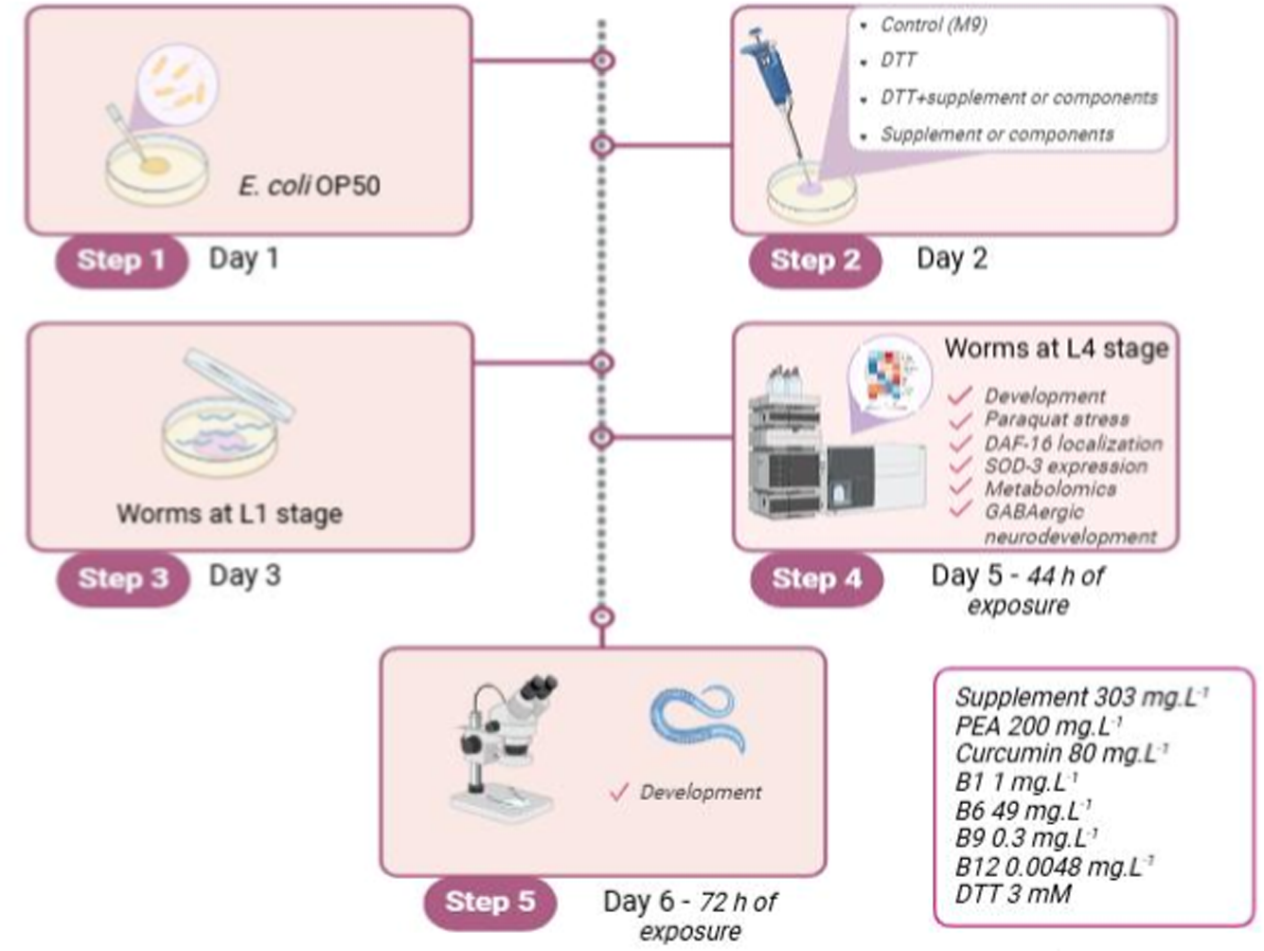
Experimental design. Step 1: NGM plates were seeded with *E. coli* OP50 and incubated overnight; step 2: the solutions (supplement 303 mg mL^–1^ or its components (PEA 200 mg mL^–1^, curcumin 80 mg mL^–1^, B1 1 mg mL^–1^, B6 49 mg mL^–1^, B9 0.3 mg mL^–1^, and B12 0.0048 mg mL^–1^) or DTT 3 mM) were added to the surface of the NGM plate seeded with *E. coli* OP50 and incubated in a refrigerator for 24 h; step 3: the synchronized worms at the L1 larval stage were transferred to the previously prepared NGM plates to the supplement or its components, with or without DTT; step 4: after 44 h of exposure, developmental analyses, resistance to paraquat stress, DAF-16 localization, SOD-3 expression, GABAergic neuron development, and metabolomics were performed; step 5: 72 h after exposure, the nematode development was analyzed.

### 2.4 Development and Body Length Measurements

Worms were exposed to the supplement with isolated compounds with or without DTT on plates. They were observed as the larval stage at 44 and 72 h at 20 °C. The worms were classified as per their stages: L1, L2/L3, L4, or adult. The worm size was assessed after image acquisition of 10 nematodes per group and quantification using ImageJ software (NIH, Bethesda, MD, USA).^30^ The experiments were carried out in duplicates on three different batches/independent experiments.^31^

### 2.5 Neurodevelopmental Assessment

GABAergic neuronal development in *C. elegans* exposed to the supplement or components was assessed using the GABAergic fluorescent strain EG1285.^32^ After treatment, nematodes were observed on the slides under a fluorescence microscope. The cell bodies of D-type GABAergic neurons were counted on the ventral cord to assess neurodevelopment after 44 h of exposure. DTT (3 mM) was used to induce GABAergic neurodevelopmental delay. The experiments were carried out in triplicate and were repeated on three separate days.

### 2.6 Stress Resistance under Paraquat

The stress resistance assay was performed using the stressor paraquat (1-1′-dimethyl-4-4′-bipyridinium dichloride). The worms (L1) were pre-exposed to the treatment and after 44 h (L4) were submitted to postexposure to 5 mM paraquat (Gramoxone 200).^33^ The protective effect of the treatments was assessed by the analysis of survival after exposure to paraquat, counted hourly for 6 h following paraquat exposure. Survival of a control group without postexposure to the stressor agent was also analyzed.

### 2.7 DAF-16 Localization

DAF-16 transcription factor was verified using a strain in which the promoter gene of this protein is tagged with a green fluorescent protein (GFP). This factor, when located in the cell nucleus, leads to the expression of detoxifying and antioxidant enzymes.^34^ TJ356 worms were exposed for 44 h to the components or supplement, with or without DTT (3 mM) coexposure, and then evaluated (L4 stage, 44 h). The worms were transferred to glass slides and covered with coverslips for imaging using a FLoid Cell Imaging Station fluorescent microscope (Thermo Fisher Scientific, Catalog number: 4471136, USA). Results were expressed as nuclear, intermediate, or cytosolic. All assays were repeated three times independently using 30 worms per group in each assay.

### 2.8 SOD-3 Expression

To verify the expression of target DAF-16 genes following exposure to the dietary supplementation, the expression of superoxide dismutase-3 (SOD-3) was evaluated by using a strain which the promoter gene is tagged with GFP.^35^ Therefore, CF1553 worms were exposed to the supplement or its components, with or without DTT (3 mM) and after 44 h coexposure worms were analyzed (L4 stage). Animals were transferred to glass slides containing levamisole (10 mM) and covered with coverslips to obtain the images using a FLoid Cell Imaging Station fluorescent microscope (Thermo Fisher Scientific, Catalog number: 4471136, USA). The head fluorescence intensity of each worm was quantified using ImageJ (NIH, Bethesda, MD, USA) software for Windows. We performed three independent assays with 10 animals in each group per independent assay.

### 2.9 Metabolomic Analysis

Approximately 10,000 worms were extracted by protein precipitation and physical disruption assisted by freeze and thaw cycles. Extracts were analyzed by two LC–MS approaches: untargeted and targeted analyses. For the untargeted analysis, we used an ACQUITY UPLC system coupled to a XEVO-G2XS Quadrupole Time-of-Flight (QToF) mass spectrometer (Waters, Manchester, UK) equipped with an ESI (electrospray ionization) system in both positive and negative ionization modes. For the targeted analysis, we used a Waters Quattro Micro-triple quadrupole mass spectrometer equipped with a Shimadzu SIL-20A LC system. Flow injection analysis (FIA) was employed, in a 4 min run, using multiple reaction monitoring (MRM) transitions for 20 amino acids and 14 acylcarnitines. More information can be accessed in the Supporting Information.

Untargeted data were processed using the Progenesis QI v2.4 software (Nonlinear Dynamics, Newcastle, UK), and the identification of the metabolites used fragmentation score, mass accuracy, mass error (precursor ions ≤ 5 ppm and fragments ≤ 10 ppm), and isotope similarity. External spectra libraries were used, such as LipidMaps (http://www.lipidmaps.org/), Human Metabolome Database (http://www.hmdb.ca/metabolites), and the MoNA—MassBank of North America (https://mona.fiehnlab.ucdavis.edu/). To increase the number of fragment matches and allow compatibility of PQI to external libraries, an in-house-developed free and open-source tool named “SDF2PQI” was used and recently detailed.^36^ For targeted analysis, TargetLynx (Waters) was used for peak integration.

The Metaboanalyst 5.0 web platform was used for statistical analysis.^37^ Data were uploaded using the area of the extracted ion chromatograms. The selection of the metabolites was based on one-way ANOVA (*p* < 0.05), followed by Tukey’s posthoc analysis (*p* < 0.05). Midlevel data fusion was used for untargeted positive and negative ionization modes. Principal component analysis (PCA), heatmaps, and pathway enrichment analysis were used as detailed in the Supporting Information.

### 2.10 Pharmacokinetic Prediction

Using SwissADME (http://www.swissadme.ch), it is possible to predict the human pharmacokinetic profile of the supplement components, including absorption, distribution, metabolism, and excretion (ADME) in silico.^38^ Since curcuminoids extracted from turmeric contain 75–80% curcumin, 15–20% demethoxycurcumin, and 4–8% bisdemethoxycurcumin, these molecules were also included in the analyses.^39^ Analysis of physicochemical properties included molecular weight, number of H-donors and acceptors, topological polar surface area, lipophilicity coefficient, and hydrophilicity coefficient.^40–42^ The SwissADME tool was used to obtain information on the likelihood of gastrointestinal absorption, the permeability of the brain–blood barrier, P-glycoprotein (P-gp) substrate, and inhibition of CYP enzymes.^38^

### 2.11 Prediction of Molecule Target Interaction and Enrichment Analysis

Predictions of the supplement components and their targets were based on SwissTargetPrediction (http://www.swisstargetprediction.ch/) for human genes.^43^ We compiled the predicted targets and submitted them to Gene Ontology and UniProt enrichment analysis using FunRich functional annotation software.^44–46^ All molecules were also individually processed through PASS Online (www.way2drug.com/passonline/) to predict their biological activities (Tables S4–S7).

### 2.12 Statistical Analysis

All experiments were performed in duplicate or in triplicate and repeated at least three times in independent experiments. Data were expressed as mean ± standard error of mean (SEM), with *p* < 0.05 considered statistically significant. The normality of data distribution was confirmed by the Shapiro–Wilk test (all p’s > 0.05). Statistical significance by one-way ANOVA, followed by Tukey’s multiple comparison test, was performed using GraphPad Prism Version 8.0.1 software. The detailed statistical data are exhibited in the Supporting Information (Tables S8–S12).

## 3 Results

### 3.1 Developmental Parameters (Larval Stage, Body Size, and GABAergic Neurodevelopment)

DTT caused a significant delay in the development of nematodes compared with the M9 control group. After 44 h (20 °C), the nematodes were expected to be in the L4 larval stage.^47^ However, animals treated with DTT were mostly in the L2 larval stage compared with the M9 control worms (Figure 2A, *p* < 0.0001). The isolated components and the supplement did not generate a delay in worms’ development compared with the M9 control group (Figure 2A). Within 72 h at 20 °C, animals are expected to reach the larval stage of gravid adult. The DTT group still showed animals in the L2 larval stage, presenting different stages compared with the M9 control group (*p* < 0.0001; Figure 2B).

**Figure 2:**
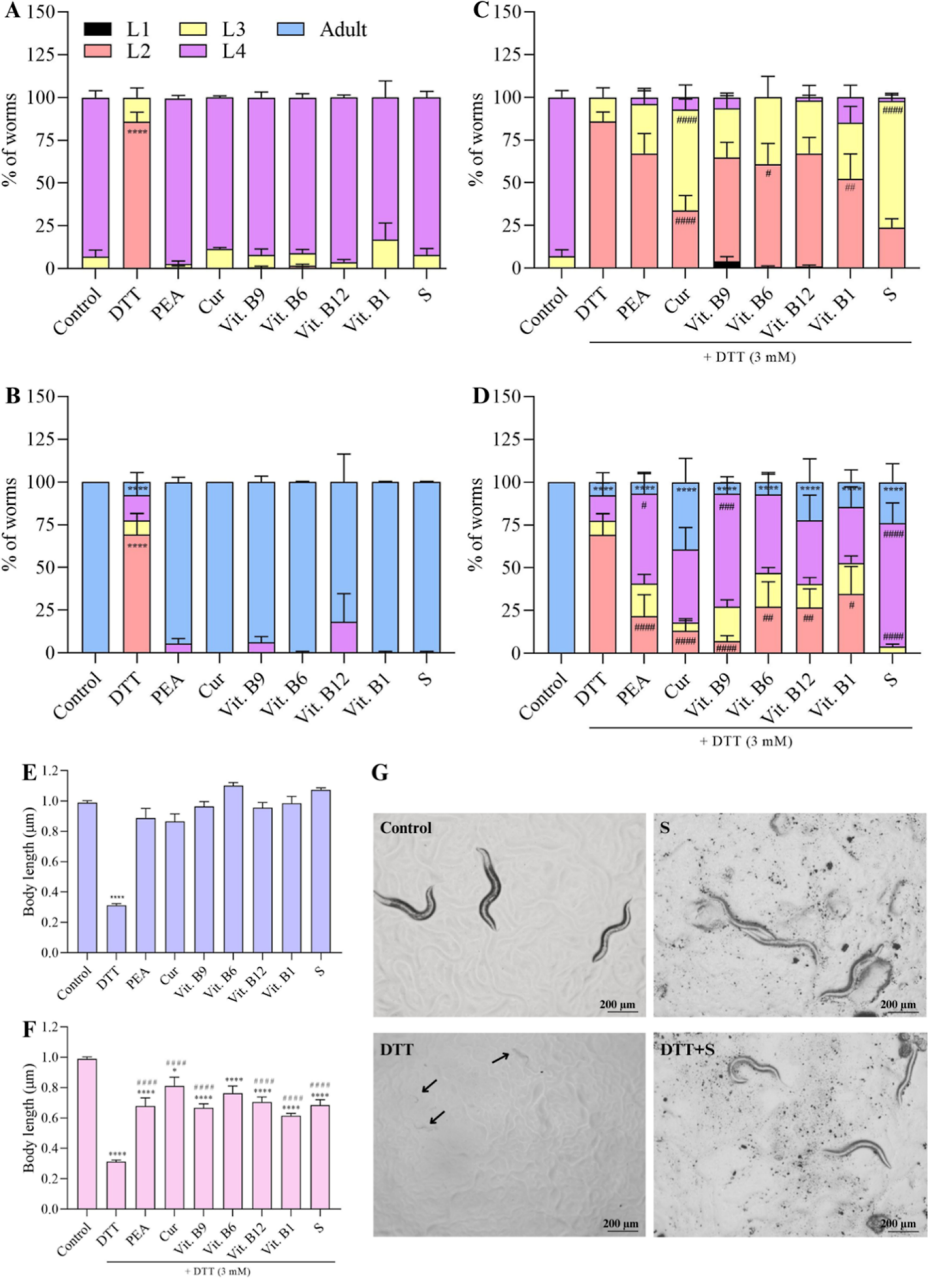
The supplement and components partially reduced the delay in worm development caused by DDT. Worms were exposed to the supplement (S, 303 mg L^–1^), PEA (200 mg L^–1^), curcumin (Cur, 80 mg L^–1^), vit. B9 (0.3 mg L^–1^), vit. B6 (49 mg L^–1^), vit. B12 (0.0048 mg L^–1^), and vit. B1 (1.0 mg L^–1^), combined or not to DTT (3 mM). The M9 buffer was used in the control groups. The percentage of worms in different larval stages (L1 stage to adult) was analyzed at 44 h (worms should achieve the L4 larval stage) and 72 h (worms should achieve adulthood). Development at isolated compounds and supplement without DTT at 44 h (A) and 72 h (B). Supplement and compounds with DTT at 44 h (C) and 72 h (D). The body length (μm) was evaluated in 44 h without (E) and with DTT (F) (*n* = 30). Representative images of treatment plates (44 h, arrow indicates worms in L2) (G). Data were expressed as the mean percentage ± standard error of the mean (SEM). Three independent assays were performed in duplicates. (*) Indicates statistically significant difference compared to the control group with *p < 0.05, **p < 0.01, ***p < 0.001, and ****p < 0.0001. (#) Denotes statistically significant difference compared to the DTT group with #*p* < 0.05, ##*p* < 0.01, ###*p* < 0.001, and ####*p* < 0.0001. The statistical analysis consisted of two-way ANOVA, followed by Tukey’s multiple comparison test.

To evaluate the effectiveness of the components or supplement in protecting against the developmental damage caused by DTT, the animals were exposed simultaneously to both DTT and the compound. After 44 h of treatment, none of the compounds tested were able to completely reverse the damage caused by DTT (Figure 2C). However, the supplement, curcumin (*p* < 0.0001), vit. B6 (*p* < 0.05), and B1 (*p* < 0.01) significantly reduced the number of animals in the L2 larval stage when compared to the DTT group (Figure 2C). Conversely, the number of animals in the L3 larval stage significantly increased in the curcumin and supplement groups (*p* < 0.0001) compared with that in the DTT group. Therefore, within 44 h, a partial reversal of the developmental delay caused by DTT was observed, particularly with curcumin and the supplement component.

After 72 h, the groups treated with DTT in combination with the supplement, PEA, curcumin, and vitamin B9 had fewer animals in the L2 larval stage compared to the DTT group (*p* < 0.0001; Figure 2D). Vitamins B6, B12 (*p* < 0.01), and B1 (*p* < 0.05) also caused a reduction in the percentage of animals in the L2 larval stage when coexposed with DTT (Figure 2D). In terms of the L4 larval stage, the groups treated with DTT together with supplements such as vit. B9 and other supplements (*p* < 0.0001), PEA (*p* < 0.001), curcumin (*p* < 0.05), vit. B6 (*p* < 0.01), and vit. B12 (*p* < 0.05), exhibited an increased percentage of animals in the L4 larval stage, thereby reducing the damage caused by DTT (Figure 2D).

The body length was reduced by DTT when compared to the control group (*p* < 0.0001, Figure 2E). However, this reduction was partially reversed by the addition of vitamins (*p* < 0.0001 compared to the DTT group, Figure 2F). Representative images illustrating the effects of DTT and DTT + S are shown in Figure 2G. Both the supplement and isolated compounds did not cause any per se effect on *C. elegans* development (Figure 2A,B,E). Therefore, while the isolated components and the supplement were partially effective in mitigating the damage caused by DTT on the developmental end points of *C. elegans*, they did not completely restore normal development.

The cell bodies of GABAergic D-type neurons were counted after 44 h of exposure (Figure 3A,B, *p* < 0.0001 compared to control). Notably, this neurodevelopmental impairment was partially restored by PEA (*p* < 0.0001), vit. B1 (*p* < 0.0001), and vit. B12 (*p* < 0.05) (Figure 3C, compared to the DTT group and M9 control group). In addition, complete protection from DTT damage was achieved with curcumin, vit. B6, vit. B9, and the supplement (Figure 3C, *p* < 0.0001 compared to the DTT group). As expected, the supplement and compounds showed no per se effects on the GABAergic D-type neuron cell bodies (Figure 3B).

**Figure 3:**
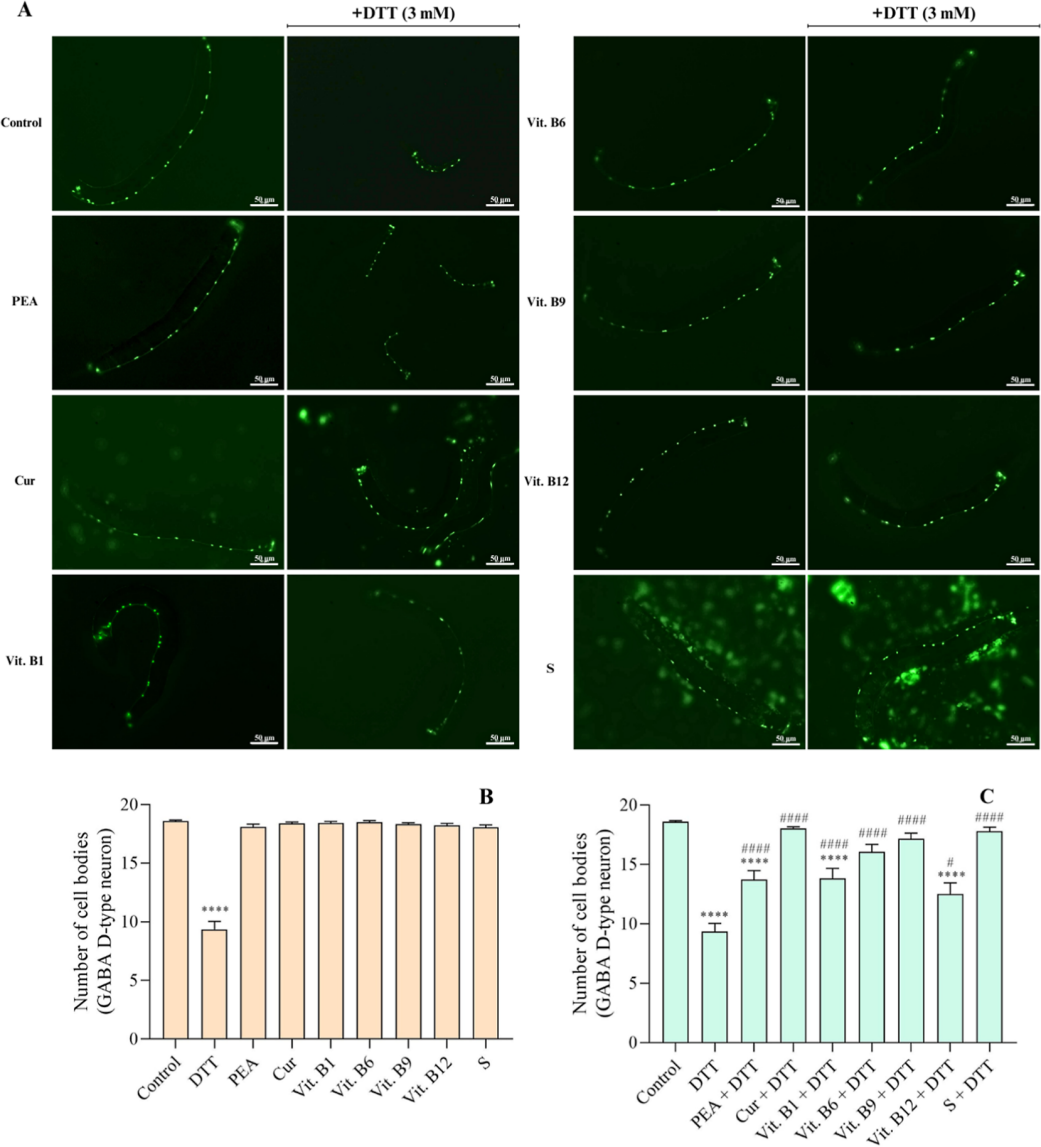
DTT impaired the GABAergic development and while the dietary supply protected the delay in *C. elegans*. Worms were exposed to the supplement (S, 303 mg L^–1^), PEA (200 mg L^–1^), curcumin (Cur, 80 mg L^–1^), vit. B9 (0.3 mg L^–1^), vit. B6 (49 mg L^–1^), vit. B12 (0.0048 mg L^–1^), and vit. B1 (1.0 mg L^–1^), combined or not to DTT (3 mM). The M9 buffer was used in the control groups. GABA D-type neuron cell bodies were quantified at 44 h of exposure at isolated compounds and the supplement without DTT (B) and supplement and compounds with DTT (C) (*n* = 40). Representative images of treatments (A). Data were expressed as mean ± standard error of the mean (SEM). Three independent assays were performed in duplicates. (*) Indicates statistically significant difference compared to the control group M9 with ****p < 0.0001. (#) Denotes statistically significant difference compared to the DTT group with #*p* < 0.05, ####*p* < 0.0001. The statistical analysis consisted of two-way ANOVA, followed by Tukey’s multiple comparison test.

### 3.2 Stress Responses

#### 3.2.1 Stress Resistance with Paraquat

Exposure to the stressor paraquat (5 mM) significantly decreased the survival rate of the worms compared to the M9 control group from the first hour onward in all situations (Figure 4A–G, *p* < 0.001). Pretreatments with PEA, curcumin, vit. B1, and vit. B6 (Figure 4A–C) did not lead a significant improvement in survival rates at any of the 4 h analyzed, when compared to the group exposed to paraquat. However, the survival rate from treatment with vit. B9 (Figure 4E) induced a significant difference in survival rates at 2 (*p* < 0.05), 3 (*p* < 0.001), and 4 (*p* < 0.05) h in comparison to paraquat. Moreover, vit. B12 treatment also protected against paraquat at 1 (*p* < 0.05), 2, 3, and 4 h (*p* < 0.001) (Figure 4F). The treatment with the supplement showed a significant reduction in mortality only at the fourth hour (*p* < 0.05, Figure 4G). Despite these protective effects, the compounds only partially mitigated the mortality caused by paraquat as they did not completely prevent it. The supplement and compounds did not affect the survival rate per se.

**Figure 4:**
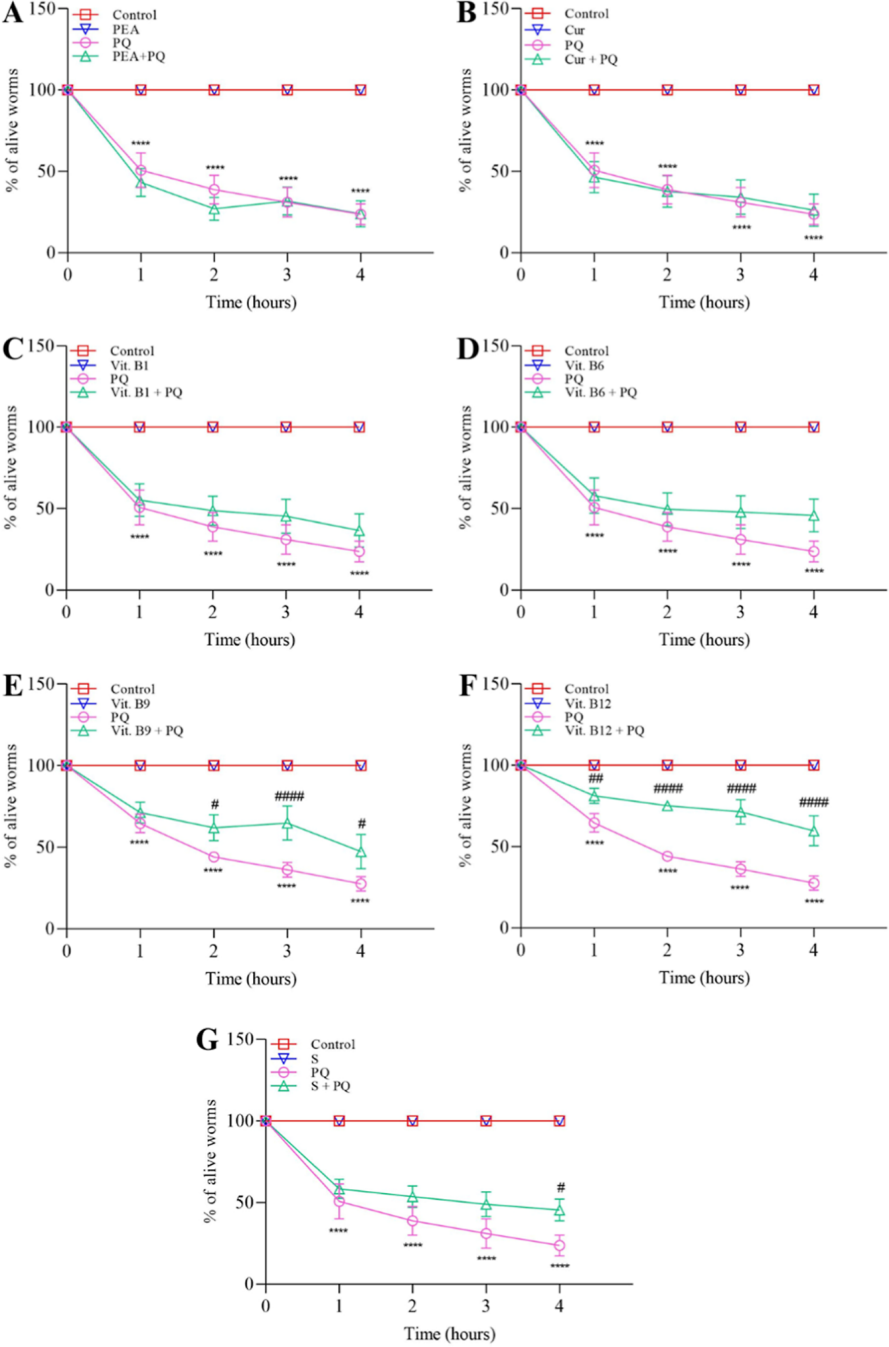
Stress resistance with Paraquat. Quantification of alive worms (L4 stage) (*n* = 8–10) after exposure to (A) PEA (200 mg L^–1^), (B) curcumin (Cur, 80 mg L^–1^), (C) vit. B1 (1.0 mg L^–1^), (D) vit. B6 (49 mg L^–1^), (E) vit. B9 (0.3 mg L^–1^), (F) vit. B12 (0.0048 mg L^–1^) and (G) supplement (S, 303 mg L^–1^) with or without paraquat (5 mM) postexposure. Data were expressed as mean ± standard error of mean (SEM). The results were analyzed by Two-way ANOVA followed by Tukey’s multiple comparison test. (*) Indicates a statistically significant difference compared to the control group M9 with ****p < 0.001. (#) Denotes a statistically significant difference compared to the Paraquat with #*p* < 0.05, ##*p* < 0.01, ####*p* < 0.001.

#### 3.2.2 DAF-16 Subcellular Localization

The migration of DAF-16 transcription factor from the cytosol to the cell nucleus is associated with stress response in *C. elegans*.^48^ Initially, we found that the components had no per se effect, as there was no significant difference when compared to the M9 control (Figure 5A–G). Subsequently, we observed that DTT significantly induced the migration of DAF-16 from the cytosol to the nucleus compared to the M9 control (Figure 5B *p* < 0.05, 5C *p* < 0.05, 5D *p* < 0.01, 5G *p* < 0.01). This suggests that the presence of this agent triggers a stress response. Furthermore, curcumin (*p* < 0.05), vit. B1 (*p* < 0.05), B6 (*p* < 0.05), and the supplement (*p* < 0.05) induced a possible protective effect against coexposure to DTT, as they reduced the migration of the transcription factor to the nucleus when compared to the positive control, maintaining levels similar to those of the M9 control (Figure 5B,C,D,G). However, no significant differences were observed between PEA, vit. B9, and B12 components when cotreated with DTT (Figure 5A,E,F).

**Figure 5:**
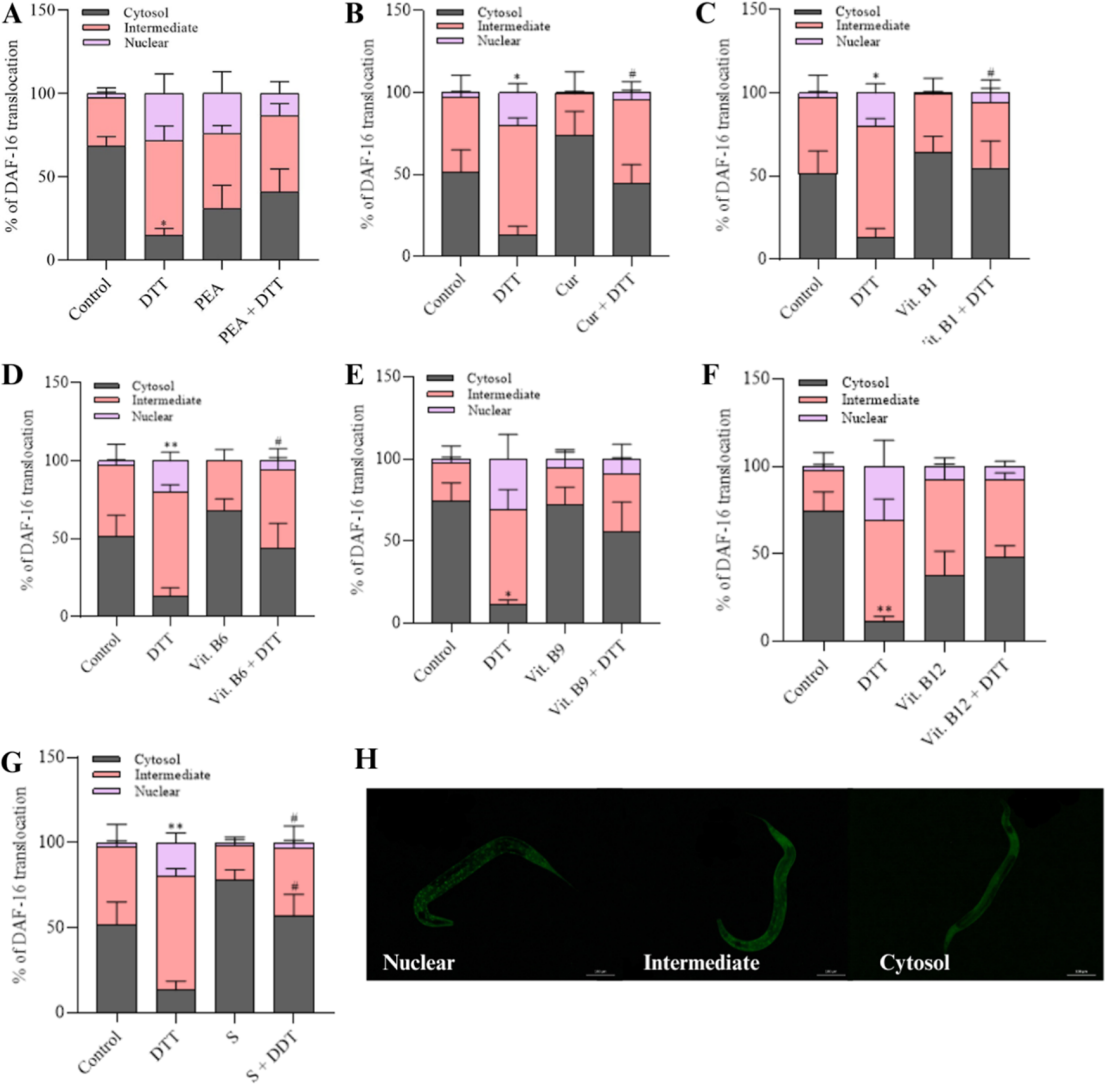
Localization of DAF-16 after exposure to the components or supplement. Cellular localization of the transcription factor *daf-16*::GFP in strain TJ356 (L4 stage) (*n* = 3–4) after exposure to (A) PEA (200 mg L^–1^), (B) curcumin (Cur, 80 mg L^–1^), (C) vit. B1 (1.0 mg L^–1^), (D) vit. B6 (49 mg L^–1^), (E) vit. B9 (0.3 mg L^–1^), (F) vit. B12 (0.0048 mg L^–1^), and (G) supplement (S, 303 mg L^–1^), with or without DTT (3 mM) coexposure. (H) Representative images of the TJ356 strain exposed to components or the supplement. Data were expressed as mean ± standard error of mean (SEM). (*) Indicates statistically significant difference compared with the control M9; *p < 0.05, **p < 0.01. (#) Denotes statistically significant difference compared with the positive control DTT; #*p* < 0.05, ##*p* < 0.01. The statistical analysis consisted of one-way ANOVA, followed by Tukey’s multiple comparison test.

#### 3.2.3 SOD-3 Expression

To evaluate the antioxidant system in *C. elegans*, SOD-3 protein expression was assessed through GFP quantification. The isolated components maintained similar expression levels to the M9 control (Figure 6A,C–F). Of note, the supplement showed a per se decrease in SOD-3 expression when compared to the M9 control, showing similar expression levels to the group exposed to DTT (Figure 6G, *p* < 0.05).

**Figure 6:**
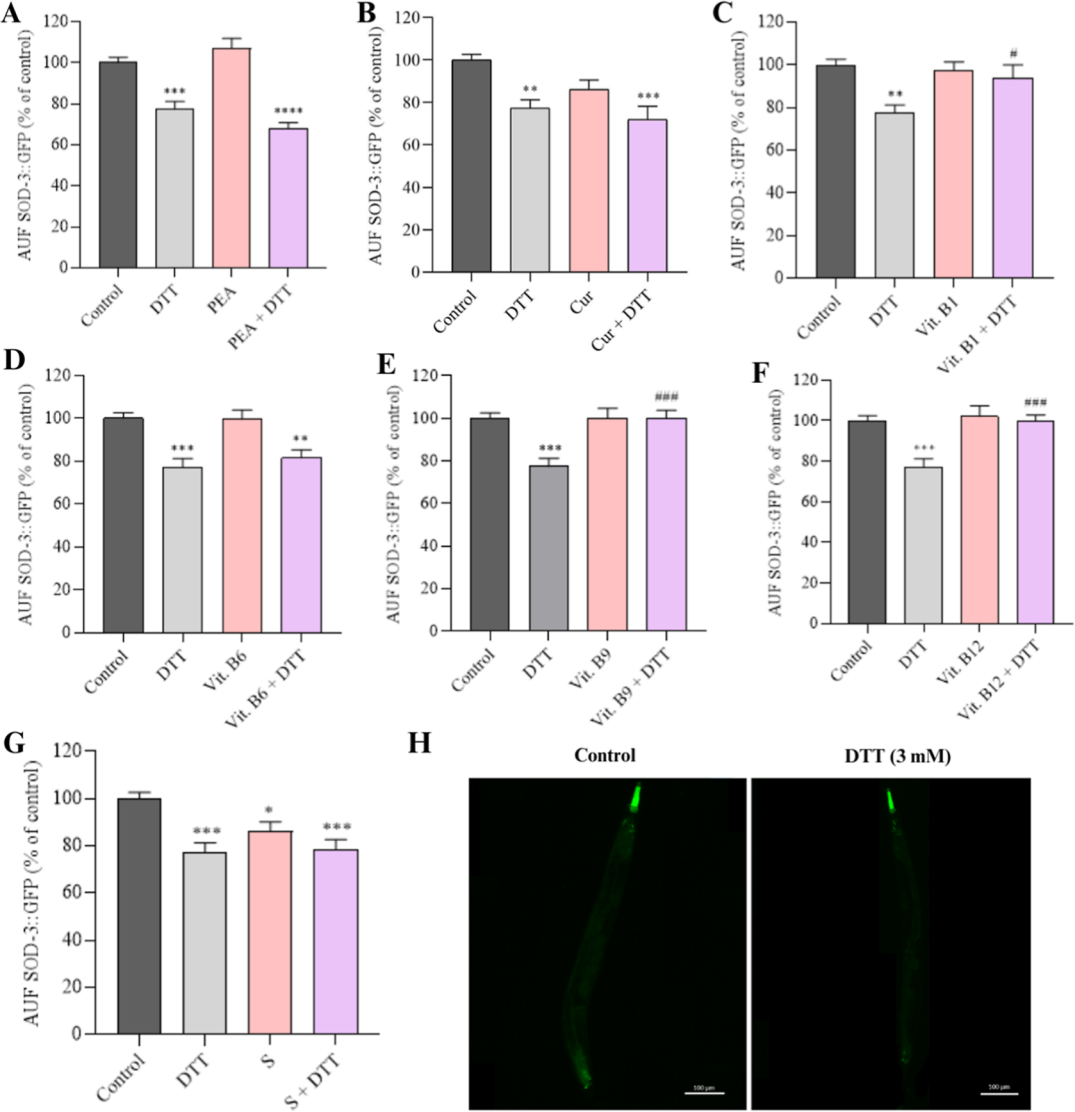
Relative fluorescence of SOD-3 after exposure to the components or supplement. (A) Quantification of *sod-3*::GFP in strain CF1553 (L4 stage) (*n* = 30) after exposure to (A) PEA (200 mg L^–1^), (B) curcumin (Cur, 80 mg L^–1^), (C) vit. B1 (1.0 mg L^–1^), (D) vit. B6 (49 mg L^–1^), (E) vit. B9 (0.3 mg L^–1^), (F) vit. B12 (0.0048 mg L^–1^), and (G) supplement (S, 303 mg L^–1^), with or without DTT (3 mM) coexposure. (H) Representative images of the CF1553 strain exposed to the components or supplement. Data were expressed as mean ± standard error of mean (SEM). (*) Indicates statistically significant difference compared with the control M9; *p < 0.05, **p < 0.01, ***p < 0.001. (#) Denotes statistically significant difference compared with the positive control DTT; #*p* < 0.05, ##*p* < 0.01, ###*p* < 0.001, ####*p* < 0.0001. The statistical analysis consisted of one-way ANOVA, followed by Tukey’s multiple comparison test.

We observed that exposure to DTT decreased SOD-3 expression in *C. elegans* (Figure 6A–G, *p* < 0.001). We found that vit. B1 (*p* < 0.05), B9 (*p* < 0.001), and B12 (*p* < 0.001) coexposed to DTT reached the levels of the M9 control, suggesting the positive modulatory effect of the vitamins on protein expression (Figure 6C,E,F). Worms exposed to the other components (PEA, curcumin, and vit. B6) and supplement coexposed to DTT did not show a significant difference in SOD-3 expression when compared to DTT (Figure 6A,B,D,G).

### 3.3 Metabolomic Analysis

Targeted analysis included 15 amino acids and 12 acylcarnitines after the removal of 7 molecules, with relative standard deviation (RSD) > 15% (Tables S1 and S2). These analytes were able to cluster the groups when a PCA score plot was created, explaining 57% of the variance of the samples (Figure S1A). In the heatmap, when comparing DTT and control groups, proline, threonine, and serine were less abundant in the control group, while arginine, C4, and C14 were more abundant (Figure 7, *p* < 0.05). When comparing DTT and supplement + DTT groups, serine, leucine, threonine, C16, methionine, and alanine were found to be more abundant in the DTT group, while arginine, histidine, proline, and lysine were found to be abundant in the supplement + DTT group.

**Figure 7:**
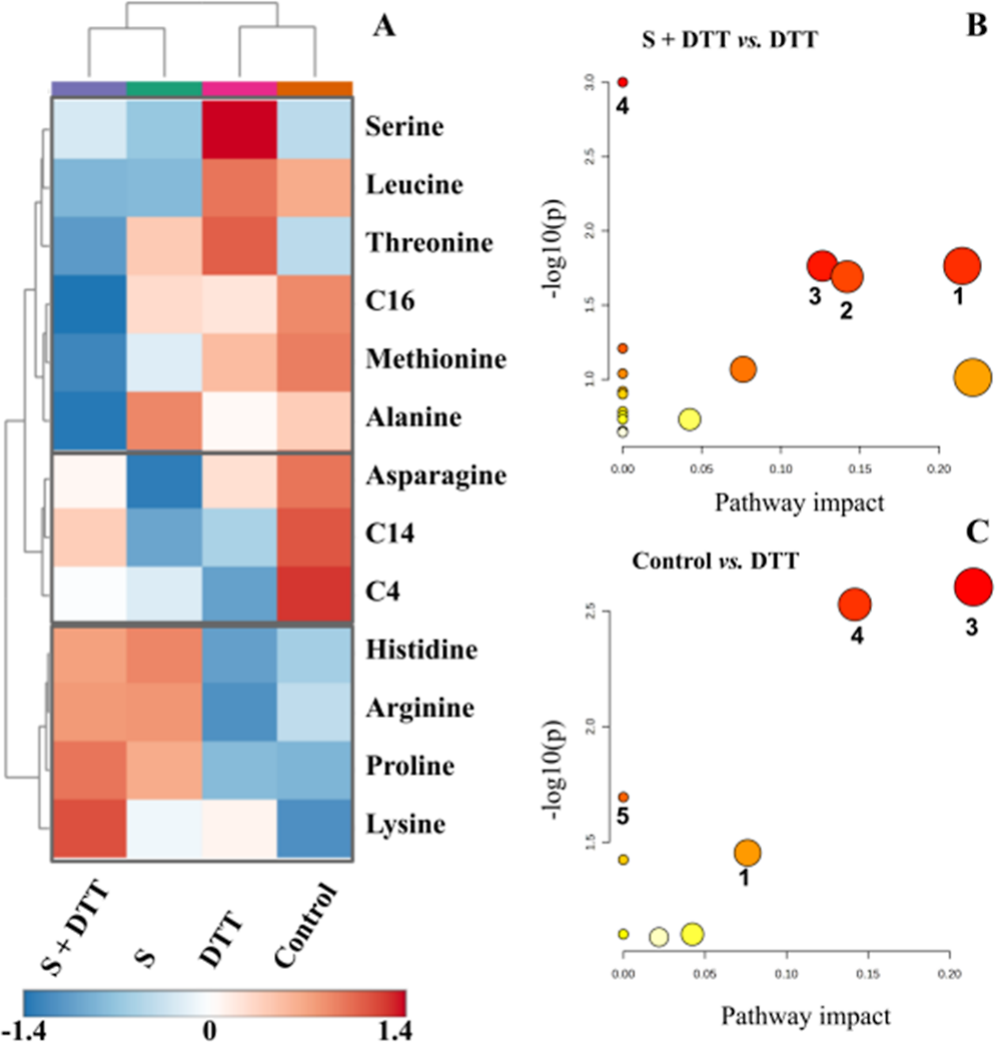
Heatmap visualization of significantly altered amino acids and acylcarnitines between the studied groups in *C. elegans*. Red color indicates high analytical signal intensity and blue indicates low intensity. Acylcarnitines are represented by their carbon number (A). Pathway analysis (*p* ≤ 0.05) based on the altered amino acids and acylcarnitines comparing the supplemented DTT group (supplement + DTT) with the DTT group (B) and the control group with the DTT group (C). ^1^Valine, leucine, and isoleucine biosynthesis; ^2^cysteine and methionine metabolism; ^3^glycine, serine, and threonine metabolism; ^4^arginine and proline metabolism; ^5^arginine biosynthesis. DTT: dithiothreitol; S: supplement.

The supplement led to an increase in arginine, histidine, lysine, and proline compared to the control group and an increase in arginine, histidine, lysine, and alanine when compared to the DTT group. Interestingly, the arginine level was reduced in the DTT group (DTT vs control) and recovered in the supplement + DTT group (supplement + DTT vs DTT). Indeed, the arginine biosynthesis pathway was found altered when comparing the control versus DTT groups (*p* = 0.035, Figure 7C) and not impacted in the comparison of supplement + DTT versus DTT (Figure 7B). A decrease in leucine, C4, C14, and asparagine levels was observed when comparing the supplemented group with the control one (supplement vs control), while a decrease in leucine, alanine, threonine, methionine, serine, and C16 was observed when comparing supplement + DTT versus DTT. Notably, threonine and serine levels were restored to lower levels in the presence of the supplement, as they had decreased when comparing the control and DTT groups.

For the untargeted analysis, a total of 1298 features were identified in negative ionization mode and 3028 in positive ionization mode after processing the raw data. The features with RSD >30% were removed, leaving 2226 for statistical analysis and midlevel data fusion. The metabolic variation between the groups was evidenced by the clustering of the groups in the PCA score plot, explaining 45.5% of the variance of the samples (Figure S1B). One-way ANOVA (*p* < 0.05), followed by Tukey’s posthoc analysis (*p* < 0.05), resulted in the detection of 839 statistically significant compounds. Among these, focus was given to compounds identified as phosphatidylcholines (PC) and lyso-PC (LPC), phosphatidylethanolamine (PE), and lyso-PE, as these compounds are known to be altered in ASD children.^49,50^

Lyso-PC and PC species (LPC 18:0, LPC 18:2, LPC 14:1, LPC 16:1, LPC 18:0, LPC 18:1, LPC 18:4, PC 12:0, and PC 22:1) were, in general, more abundant in the DTT group compared to the control group, while only three PC presented the opposite trend (LPC 16:0, LPC 20:5, and PC 14:0) (Figure 8). For the PE class, 15 species were detected, of which 9 were lower in the DTT group than in the control (lysophosphatidylethanolamine (LPE) 14:0, LPE 16:1, LPE 18:0, LPE 18:1, LPE 20:0, LPE 20:1, LPE 20:5, PE 16:0, and PE 30:5), and 3 showed the opposite trend LPE 18:3, PE 16:1, and PE 22:3 (Table S3).

**Figure 8:**
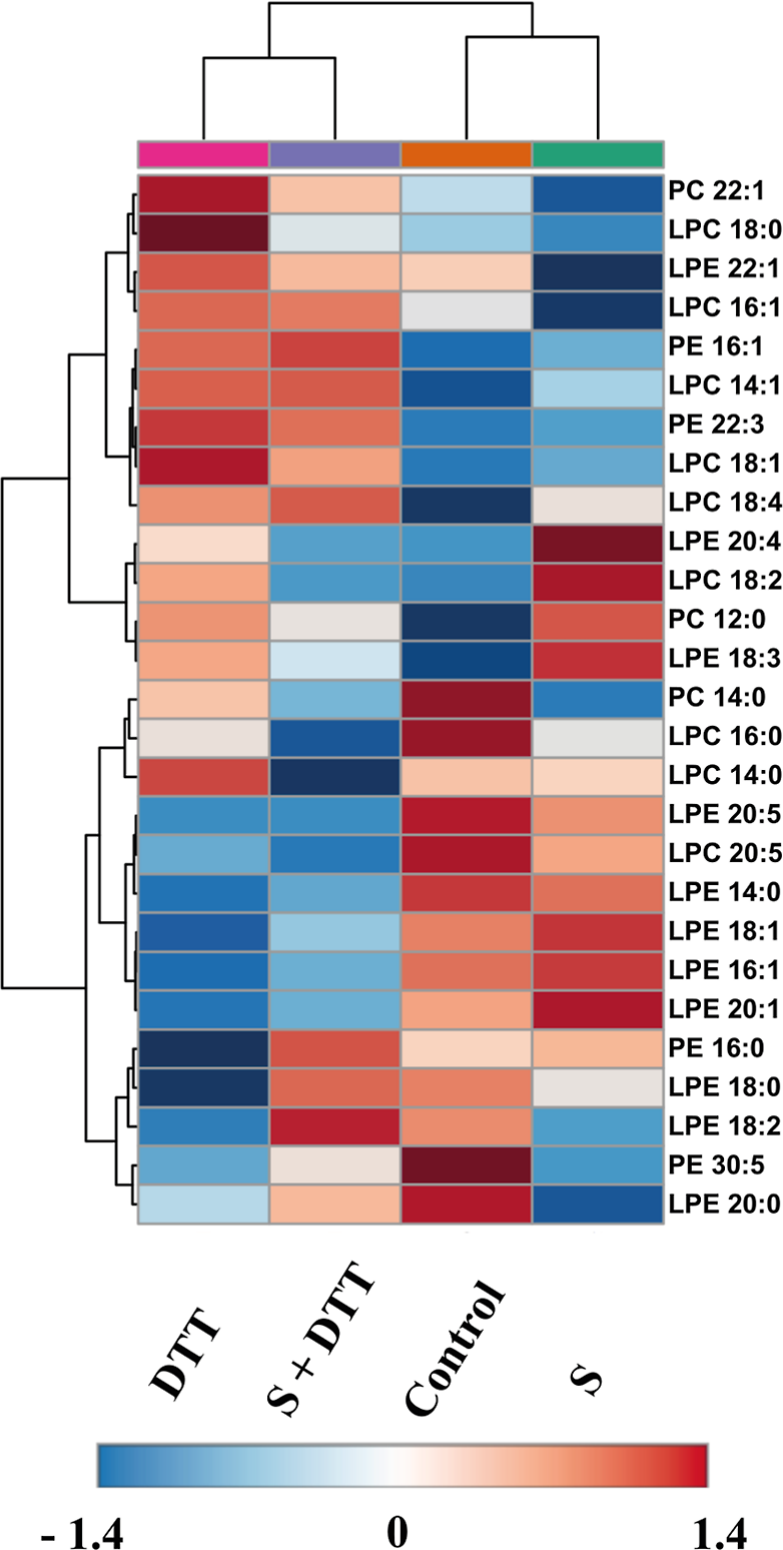
Heat map visualization of significantly altered LPC, PC, LPE, and PE between the studied groups. Red color indicates high analytical signal intensity, and blue indicates low intensity. LPC: lysophosphatidylcholine; PC: phosphatidylcholine; LPE: lysophosphatidylethanolamine; PE: phosphatidylethanolamine; S: supplement; DTT: dithiothreitol.

When examining the effect of supplementation on the overall PC and PE species profile, some specific trends were noted for PC species (LPC 14:0; LPC 18:2; LPC 18:0; LPC 18:1; PC 22:1; and PC 12:0) that had their levels reduced in the DTT group (DTT vs control) and increased after supplementation (DTT vs supplement + DTT). Increased levels of LPE 18:0 and PE 16:0 were observed after supplementation (DTT vs supplement + DTT).

### 3.4 Computational Analysis

#### 3.4.1 ADME Properties

The prediction of the pharmacokinetics for the compounds is shown in Table 1. However, due to the size of the molecule, SwissADME was unable to process results for vit. B12. As expected, the analyzed vitamins have log *S* compatibility with hydrosoluble molecules higher than −4.^38,40^ These results present data compatible with the high hydrophobic profile of PEA and curcuminoids (log *P*s greater than −4), which can negatively impact their bioavailability.^38^

**Table 1:** SwissADME Numerical Results^a^

molecule	MW (g mol^–1^)	H-bond donors	H-bond acceptors	Mlog*P* (o/w)	log *S* (Ali)	Wlog*P* (o/w)	TPSA (Å^2^)
PEA	299.49	2	2	3.39	–7.00	4.58	49.33
curcumin	368.38	2	6	1.47	–4.83	3.15	93.06
demethoxycurcumin	338.35	2	5	1.80	–4.76	3.14	83.83
bisdemethoxycurcumin	308.33	2	4	2.13	–4.50	3.13	74.60
vitamin B1 (thiamine)	265.35	2	3	0.05	–2.80	0.62	104.15
vitamin B6 (pyridoxine)	169.18	3	4	–0.91	–0.30	–0.22	73.58
vitamin B9 (methylfolate)	441.40	6	9	–0,62	–2.91	–0.38	213.23

a MW: molecular weight; TPSA: topological polar surface area.

Table 2 indicates that PEA and curcuminoids have the potential to inhibit CYP enzymes, specifically, CYP1A2, CYP2C9, CYP2D6, and CYP2A4. Among all the tested components, vit. B9 is expected to have low gastrointestinal absorption. Curcumin, demethoxycurcumin, and vit. B1, and vit. B9 are not predicted to be permeable through the brain blood barrier (BBB). Vitamin B1 is also predicted to be a P-gp substrate. The prediction for PEA and curcuminoids shows inhibition of CYP enzymes.

**Table 2:** SwissADME Nominal Results^a^

molecule	GI absorption	BBB permeant	P-gp substrate	CYP1A2 inhibitor	CYP2C19 inhibitor	CYP2C9 inhibitor	CYP2D6 inhibitor	CYP3A4 inhibitor
PEA	high	yes	no	yes	no	no	yes	no
curcumin	high	no	no	no	no	yes	no	yes
demethoxycurcumin	high	no	no	yes	no	yes	no	yes
bisdemethoxycurcumin	high	yes	no	yes	no	yes	no	yes
vitamin B1 (thiamine)	high	no	yes	no	no	no	no	no
vitamin B6 (pyridoxine)	high	no	no	no	no	no	no	no
vitamin B9 (methylfolate)	low	no	no	no	no	no	no	no

a GI: gastrointestinal; BBB: brain blood barrier; P-gp: P-glycoprotein.

#### 3.4.2 Enrichment Analysis

The Reactome pathways are shown in Figure S2 based on UniProt enrichment analysis conducted through FunRich. Reversible hydration of carbon dioxide, prostanoid ligand receptors, G-alpha (q) signaling, interleukin 4 and 13 signaling, G-alpha (i) signaling, nuclear receptor transcription pathway, EPH-Ephrin signaling, PIP3-activated AKT signaling, VEGFR2-mediated cell proliferation, and muscarinic acetylcholine receptors are presented to be involved in the Reactome pathway related to these molecules’ target predictions.

## 4 Discussion

According to the Center for Disease Control and Prevention (CDC), the prevalence of ASD in the population has reached 1%.^3^ The mechanisms that trigger ASD are complex, and no specific pharmacological treatment for core symptoms is available. This has led to the emergence of new possibilities for management. Currently, the most common methods of support for ASD include occupational, behavioral, speech, and play therapies. Although drugs such as antidepressants, anticonvulsants, antipsychotics, and stimulants have been used to treat ASD symptoms, none of them have been able to fully reverse the main symptoms. Additionally, these drugs can cause important side effects.^51^ On the other hand, natural products and dietary supplements, such as vitamins and minerals, have been suggested as potential treatments for various conditions, including ASD and other neurodevelopmental disorders.^51^

For ASD research, another crucial limitation is the reduced availability of animal models to investigate new treatments. Here, a new model is proposed for fast testing of a new approach to induce some features and metabolic markers of an ASD endophenotype: the nematode *C. elegans* exposed to DTT. DTT is a demethylating and stress-inducing agent, a chemical model produced in *C. elegans-*specific biochemical and molecular changes present in some ASD patients, especially changes in enzymes that catalyze methylation, such as methionine synthase, resulting in developmental delays in the nematode.^27^ This model may help us understand the potential association between altered methylation and ASD. DTT exposure triggers specific responses, activating the hypoxia response pathway and modulating the methionine–homocysteine cycle. The induction of the hypoxia response pathway, mediated by the hypoxia-inducible factor (HIF-1), indicates DTT as a stressor by mimicking low-load conditions. DTT influences the metabolic cycle by altering the SAM levels and upregulating the SAM-dependent methyltransferase gene, *rips-1*. These changes impact *C. elegans* growth, development, and life cycle transitions, revealing the crucial role of DTT as a reducing agent in model organisms.^52^ Our results corroborate the literature once DTT also caused a delay in *C. elegans* larval progression and body size. In agreement, DTT delayed and impaired the GABAergic neuron development. In *C. elegans*, there are 19 ventral cord D-type GABA motor neurons whose function is to inhibit contraction of the body wall muscles during movement, being crucial for the animals’ survival (food foraging and escaping from adverse environments).^53^

Alterations in the GABAergic system have been linked to the development of both genetic and nongenetic endophenotypes of ASD in humans, although the precise molecular mechanisms remain unclear. In children with ASD, GABAergic dysfunction has been associated with impairments in cognitive processes such as somatosensory integration, attention, and social behavior.^54^ This notion is further supported by studies in other animal models of ASD, which demonstrate compromised GABAergic and glutamatergic neurotransmission.^55^ Additionally, lower GABA levels and functional changes in GABAergic receptors have been implicated in ASD pathophysiology.^55^

Conversely, increased glutamate levels have been observed in postmortem and body fluid samples from both pediatric and adult patients with ASD. Dysregulation of glutamatergic signaling, similarly documented in ASD patients and animal models, is thought to contribute to the higher prevalence of epilepsy in this population, given the critical role of glutamatergic pathways in seizure activity.^56^

The disruption of GABAergic and glutamatergic signaling creates a significant E/I imbalance, which underlies the core symptoms of ASD, including social difficulties, repetitive behaviors, and heightened susceptibility to epilepsy.

Another etiopathological mechanism of ASD that has been evidenced in our DTT model is the impairment of the antioxidant response. Numerous studies have indicated the presence of oxidative stress in individuals with ASD, which implicates a possible pathogenesis mechanism.^57^ Thus, an imbalance in oxidative metabolism may contribute to ASD by causing oxidative damage during embryonic and fetal development as well as in early childhood, ultimately leading to neurodegeneration. Children with ASD exhibit impaired endogenous antioxidant defenses, including reduced total GSH levels and altered activities of key enzymes such as GPx, SOD, and CAT.^58^ From this perspective, the use of supplements that enhance the activity of these enzymes could serve as a potential strategy to reduce the incidence of new ASD cases or as a therapeutic option for affected individuals. Our findings demonstrated that the supplement, along with some of its isolated components, significantly decreased the mortality rate induced by the pro-oxidant paraquat in nematodes. Furthermore, it modulated the nuclear migration of transcription factor DAF-16, contributing to the prevention of stress responses triggered by DTT.

PEA is known for its benefits against neuroinflammation and neurodegeneration caused by glutamate.^20,59^ It is already used in some European countries as a supplement due to its antioxidant and neuroprotective properties.^60^ PEA is now clearly related to the endocannabinoid system, sharing metabolic pathways and targets that help maintain body homeostasis.^61^ In the context of ASD, PEA has improved irritability and hyperactivity in children.^59^ In the *C. elegans* model, PEA partially recovered the development and GABAergic neurodevelopment delay caused by DTT, likely via mechanisms other than affecting antioxidant responses. Our study also demonstrates that PEA treatment increases the expression of the antioxidant enzyme SOD-3, indicating its ability to stimulate this important protective enzyme.

Curcumin, an active compound in turmeric (*Curcuma longa*), shows promise as a treatment for ASD in animal studies.^62^ Curcumin’s antioxidant and anti-inflammatory properties may benefit individuals with ASD. When DTT toxicity was induced, larval development experienced partial protection, while curcumin completely restored GABAergic neurodevelopment. Moreover, it re-established the DAF-16 transcription factor localization to the control levels, possibly through this pathway, indicating that it can modulate antioxidant responses and *C. elegans* development. A study conducted on *C. elegans* examined the effects of curcumin supplementation, revealing that it enhances the longevity of these nematodes by upregulating antioxidant genes such as *sod-1*, *sod-2*, and *sod-3*.^63^ Moreover, another study highlighted curcumin’s role in aging due to the activation of antioxidant enzymes such as SOD in rats.^64^ These evidence support our results, which show that curcumin not only increased the nuclear migration of DAF-16 but also elevated the expression of SOD-3 when coexposed with DTT.

Vitamin B1 is a trace element that is important to carbohydrate metabolism and energy production. Its role in transporting and eliminating heavy metals in the body prevents damage from these toxicants. Additionally, its neuroprotective properties warrant further exploration.^64^ Some studies suggest that a deficiency in vit. B1 may lead to neurological disturbances, including ASD.^65,66^ In *C. elegans*, vit. B1 supplementation can improve mitochondrial stress and enhance survival rates.^67^ Under DTT exposure, vit. B1 partially mitigated development delays and increased body length, restored DAF-16 translocation, and increased the expression of SOD-3 in the nematodes. The antioxidant activity of vit. B1 by the increase of SOD and CAT activities has already been reported. Therefore, the deficiency of this vitamin contributes to oxidative stress and decreases the activity of these enzymes.^68^ The relation of vit. B1 with the GABAergic system is vital in neuronal development and modulation of excitatory neurotransmission impacting GABA and glutamate activities, and the perinatal deficiency of this vitamin leads to disruption of the neuromotor behavior and decrease in the concentration of GABA and glutamate.^69^ Conversely, in our study, vit. B1 could not rescue the GABAergic damage in *C. elegans* treated with DTT, probably due to the low concentration used.

Vitamin B6 can positively affect ASD individuals without causing notable side effects. Studies have shown that vit. B6 treatment can lead to a decrease in behavioral problems by improving appropriate behavior and brain wave activity.^70^ It is worth noting that vit. B6 is crucial for the synthesis of several neurotransmitters such as GABA, noradrenalin, serotonin, histamine, dopamine, glycine, and d-serine. This implies that vit. B6 supplements could potentially enhance multiple neurotransmitter systems.^71^ In experiments with *C. elegans*, supplementation with vit. B6 was linked to an extended lifespan.^72^ Here, vit. B6 ameliorated the development and GABAergic delay, rescued the DAF-16 transition to control levels, and increased the expression of SOD-3 in relation to the positive control DTT, returning its levels the same as the control. Our findings are consistent with the literature, as many studies have reported that vit. B6 fulfills antioxidant goals by quenching ROS.^73^

Vitamin B9 (folate) is an essential micronutrient that helps prevent congenital disabilities, particularly neural tube defects. It is necessary for forming several coenzymes in many metabolic systems, including purine, pyrimidine, and nucleoprotein synthesis, as well as maintaining erythropoiesis. Therefore, its deficiency may lead to neurological symptoms in ASD.^74,75^ Vitamin B9 treatment protected the mice from paraquat stress and improved DTT-induced development delay, GABAergic neurodevelopment, and SOD expression. In *C. elegans*, vit. B9 has been reported for increasing oxidative stress resistance and longevity by elevating the expression levels of relevant genes in stress response and lifespan, including *daf-16*, *skn-1*, and *sir 2.1*, as well as glutathione *S*-transferase 4 (GST-4) and SOD-3 enzymes.^76^

Vitamin B12 is a water-soluble essential micronutrient implicated in two main metabolic pathways: the canonical propionate breakdown pathway and the methionine/S-adenosylmethionine (Met/SAM) cycle. In *C. elegans*, low levels of vit. B12 or genetic disruption of the canonical propionate breakdown pathway lead to an accumulation of propionate and the transcriptional activation of a propionate shunt pathway.^77^ Vitamin B12 can be transported by the ABC transporter *mrp-5* from the intestine to support *C. elegans’* embryonic development.^78^ In addition, vit. B12 could alleviate the development delay induced by DTT in *C. elegans*.^79^ Our results also demonstrated that supplementation with vit. B12 protects the nematodes from stress induced by paraquat; however, it does not show antioxidant activity in the studied pathways. The deficiency of vit. B12 can cause cellular accumulation of ROS and nitric oxide and decrease in antioxidants and CAT/SOD activity, resulting in oxidative damage and memory impairment in worms.^80^ Even though our results did not indicate a significant increase in the expression of SOD or modulation of the nuclear translocation of DAF-16, it is essential to note that vit. B12 may still play a crucial role as a neuroprotective agent against ASD. Finally, vit. B12 provided partial protection against GABAergic impairment caused by DTT, underscoring its potential as a beneficial treatment option.

Notably, the supplement showed the best performance in improving end points compared to all the isolated compounds tested. When exposed to the supplement, *C. elegans* showed significant improvement in the antioxidant responses, development, and GABAergic neurodevelopment after DTT damage, which can be attributed to the combination of benefits of each component in the supplement. The findings support the benefits of combined supplements in humans and lead to the necessity of further studies targeting ASD children.

To enhance the agility and accuracy of biological information processing prior to in vivo testing,^81^ this study applied bioinformatics. Therefore, as there is an intention to apply this supplement to humans, it is essential to consider these molecules’ pharmacokinetics and biological properties that can affect their physiological functions. Vitamin B1, as predicted through SwissADME, is a P-gp substrate, which correlates with scientific literature that shows the cells prevent its accumulation by pumping it out.^82^ Vitamin B9 is predicted to have low GI absorption; however, the human GI tract has a carrier-mediated transport mechanism to absorb it.^83^

The current research predicted that curcuminoids can inhibit CYPs. Curcumin can inhibit CYP enzymes, specifically CYP2C9 and CYP3A4 enzymes, through noncompetitive and competitive inhibition, respectively.^84^ In vivo studies have further confirmed that curcuminoids can inhibit CYP enzymes.^85–87^ This raises concerns regarding the concurrent use of this supplement with other drugs that are metabolized by the same enzymes, such as aspirin, ibuprofen, and other nonsteroidal anti-inflammatory drugs.^88,89^

PEA- and curcumin-poor water solubility are known to disfavor their bioavailability in the bloodstream.^90,91^ Therefore, to enhance their unfavorable pharmacokinetic properties, the supplement contains PEA and curcumin with LipiSperse technology (commercially referred to as Levagen plus and Hydrocure, respectively). These new formulations promote improved absorption of PEA and curcumin in the gastrointestinal tract.^92,93^

Enrichment analysis predicted that this supplement targets pathways associated with neurodevelopmental impairment that are observed in ASD. The prediction suggests that some pathways are related to immunomodulation, such as prostanoid ligand receptors and interleukin 4 and 13 signalings. Prostanoids and interleukins play pivotal roles in immune modulation, which is a critical factor in ASD development, especially triggered by neuroinflammation dysfunction.^94^ Additionally, prostanoids, especially prostaglandin E2, are also crucial regulators of synaptic plasticity in the central nervous system and might be significant factors in ASD pathogenesis.^95^

EPH-Ephrin signaling and muscarinic acetylcholine receptors demonstrated in our results to be pathways related to the supplement’s activity; although not clearly associated with immunomodulation, these pathways also show an association with ASD in in vivo animal models through different mechanisms. In mutant mice, depletion of EPH receptors results in autistic-like phenotypes, which can be reduced by artificially activating the remaining EPH receptors and rescuing synaptic pruning.^96^ The muscarinic acetylcholine receptor regulates complex behaviors. Consequently, their agonists have been shown to reduce the restrictive repetitive behavior in the animal models of ASD.^97–99^ In relation to VEGFR2, the connection to ASD remains unclear. However, recent studies indicate a potential relationship due to the role of VEGFR2 in cerebrovascular modulation.^100–102^ In addition, enrichment analysis revealed additional pathways that have not been described yet for ASD or neurodevelopment.

In silico analysis pointed out that PEA and the curcuminoids present some pharmacokinetic properties that are unfavorable to their use as oral drugs. The tested supplement effectively addresses the problem of hydrophobicity by utilizing the LipiSperse technology. In addition, the activity of the supplement components has been predicted to positively affect some pathways involved in ASD pathogenesis, such as immunomodulation, synaptic pruning, and regulation of complex behaviors. The relation between these pathways suggests that the supplement may be a potential complementary strategy for managing ASD.

Metabolomic data in ASD patients are rare and conflicting due to the heterogeneity of the spectrum. In *C. elegans*, experiments can be controlled, and uniformity can be guaranteed. Some changes in the amino acid levels in patients with ASD have been reported. However, the profile identity is variable between different studies.^103^ Proline has been found to increase in ASD patients. This increase is hypothesized to be related to the low-activity alleles of catechol *O*-methyltransferase (COMT) and proline dehydrogenase (oxidase) 1 (PRODH), enzymes involved in the degradation of proline.^104^ Serine also has been reported as increased in ASD patients.^105^ Despite DTT-increased threonine levels, ASD patients present lower levels of this amino acid.^106^ In addition, lower levels of threonine and serine were re-established by the supplement. Although arginine was found to be reduced in the DTT group, studies on ASD children have shown that this amino acid is increased.^107^

Regarding lipid metabolism, DTT reduced the acylcarnitines (C14) in *C. elegans*, which are crucial for mitochondria for β-oxidation and healthy brain development.^108^ Carnitine biosynthesis defects have been linked to an increased risk for ASD among males.^109^ The supplement was effective in increasing C14 levels in the worms. Studies have found that children with ASD have decreased levels of PE in their erythrocyte membranes.^49^ The present study also found some of those species following the same profile of ASD children. Reduced levels of PC and lyso-PC, which are involved in glycerophospholipid metabolism, could impact language ability in children with ASD.^50^ Metabolomic analysis showed that at least three species of PC were reduced. DTT can cause the increase in SAM-dependent methyltransferase, while induces SAM depletion.^27^ This could impact the metabolism of choline and methionine, verified in the metabolomic analysis by DTT modulation on their probably related products (PC and PE). Additionally, phosphatidylethanolamine methyltransferase also utilizes SAM to convert PE to PC through methylation activity in the brain.^110^

Our study brings relevant findings on a new model for exploring therapeutic strategies for an ASD subgroup and proposes a safety management approach for the condition. However, it is essential to carefully analyze these results and recognize the limitations inherent in any experimental model. In this case, the DTT model addresses some of the known and still undisclosed mechanisms of ASD. Behavioral observations are still challenging due to the heterogeneity of larval stages, which makes the individual picking of worms required for some tests unfeasible. Conversely, it provides a rapid method for screening new drugs and advancing precision medicine approaches, which are essential given the heterogeneity of ASD pathophysiology and the increasing prevalence of diagnoses within the spectrum.

Regarding the supplement, we focused on achieving the highest possible concentration of dispersion while maintaining the same concentration ratios as those found in the supplement. Therefore, some vitamins were tested in concentrations lower than those supplied to worms by other authors (i.e., vit. B12 at 0.2 mg L^–1^,^111^ while in our study, 0.0048 mg L^–1^). This difference might explain why we did not observe some of the effects of the isolated components.

Moreover, the multiple mechanisms involved in the development of ASD can contribute to heterogeneous responses to alternative therapies, which may be implicated in the inefficiency of vitamins and natural products for some individuals. However, this variability also occurs with established drug therapies, which can often be more toxic. Therefore, it is essential to consider vitamins and natural products as potential initial alternatives for treatment.

## 5 Conclusion

Here, we present a novel chemical model using the nematode *C. elegans*, considering some characteristics of ASD, to seek both mechanisms and management strategies. DTT delayed worm development, impaired GABAergic morphology, and altered the biochemical profile related to amino acids, carnitines, and lipids. All of these specific metabolic vulnerabilities within the methylation endophenotype can be observed in a group of ASD patients. The newly tested supplement has proven to be profitable in addressing the development and stress associated with the metabolic vulnerabilities of some ASD endophenotypes. Bioinformatic analysis predicted PEA and the curcuminoid’s hydrophobic properties, which can be enhanced using LipiSperse technology in the supplement. In addition, the components present in the supplement can interact with certain human pathways associated with ASD, such as prostanoid and EPH-Ephrin signaling. Considering all of the outcomes presented in this study, the tested supplement may be a potential complementary strategy in managing ASD. Furthermore, additional research could be useful in consolidating the DTT model in studying ASD and other environmental endophenotypes of neurodevelopmental delays. Since the metabolomic analysis has uncovered several avenues to be pursued regarding important pathway changes affected by this chemical, the DTT model offers a promising model for further investigation.

## Supplementary Material



## Notes

Supplementary methods; additional metabolomic data; statistical tests and analyses related to metabolomics; prediction data of biological activities for compounds present in the supplement; statistical data from development assays in *C. elegans*; PCA plots of metabolomic analyses; and results from functional enrichment analysis (PDF)

## Abbreviations

ADME: absorption, distribution, metabolism, and excretion
AKT: protein kinase B
ASD: autism spectrum disorder
C14: acylcarnitines
CDC: Centers for Disease Control and Prevention
COMT: catechol *O*-methyltransferase
DTT: dithiothreitol
ESI: electrospray ionization
EPH: erythropoietin-producing human hepatocellular
FIA: flow injection analysis
GFP: green fluorescent protein
GI: gastrointestinal
GST: glutathione *S*-transferase
HIF-1: hypoxia-inducible factor-1
HMOX1: heme oxygenase 1
JAK2: Janus quinase 2
LPE: lysophosphatidylethanolamine
LPC: lyso-PC
MET: methionine
MMP9: matrix metalloproteinase-9
MRM: multiple reaction monitoring
MW: molecular weight
NGM: nematode growth medium
PC: phosphatidylcholines
PCA: principal component analysis
PE: phosphatidylethanolamine
PEA: palmitoylethanolamide
P-Gp: P-glycoprotein
PIP3: phosphatidylinositol (3,4,5)-trisphosphate
PRODH: proline dehydrogenase (oxidase) 1
QC: quality control
QToF: quadrupole time-of-flight
ROS: reactive oxygen species
RSD: relative standard deviation
RT: chromatographic retention time
S: supplement
SAM: *S*-adenosylmethionine
SOD-3: superoxide dismutase-3
TNF: tumor necrosis factor
TPSA: topological polar surface area
VEGFR2: vascular endothelial growth factor receptor 2
