# Supplementary material for: Nutritional Supplementation Benefits in Caenorhabditis elegans under Developmental Disruption and Stress Conditions
Source: ACS Omega. 2025 Jul 17;10(29):31313–30. doi: 10.1021/acsomega.4c10748 (PMC12311710; doi:10.1021/acsomega.4c10748)
Supplement: Supplementary file 1 [file ao4c10748_si_001.pdf]

Supplementary Materials

Nutritional supplementation benefits in *C. elegans* under developmental disruption and stress conditions

Daiana Silva Ávila[1,2], Camila Milagres Macedo Pereira[3], Iverson Conrado Bezerra[3,4], Gabriel Pedroso Viçozzi[1], Alex Ap. Rosini Silva[5], Artur José da Silva[3], Heloísa Aiolfi Padilha[2], Emilly de Souza Cordeiro[3], Aline Castro Silva[2], Danilo Cardoso de Oliveira[5], Katarine Gabriely Aurista do Nascimento[3], Julianne de Santana Cavalcante[3], Clarice Beatriz Gonçalves Silva[3], Roberto Afonso da Silva[3], Matheus Chimelo Bianchini[6], José Luiz de Lima Filho[3,4], Andreia M. Porcari[5], Priscila Gubert[3,4,7*]

Corresponding author: Priscila Gubert. priscila.gubert@ufpe.br
E-mail address: priscila.gubert@ufpe.br (Priscila Gubert)

1. *Supplementary Methods*

1.1. Metabolomics (Detailed methodology)

The worms (approximately 10.000) were extracted by adding cold methanol (500 µL). Afterward, the tubes were vortexed (30 s) and subjected to three cycles of freeze & thaw in liquid nitrogen, aiming at their physical rupture. Samples were then subjected to an ultrasound bath (10 min) vortexed for 30 s, and centrifuged (12,000 RPM, 10 min, 4 °C). The bottom organic layer (400 µL) was collected and dried in N2 flow. Samples were resuspended in a solution of 150 µL ACN: H2O (1:1, % v/v) and kept for both untargeted and targeted analysis.

Each resuspended sample (25 µL) was collected to compose a pooled sample used as the Quality Control (QC). QC samples were inserted after 3 samples each to check deviations in extraction and system stability. Sample extraction and analysis were conducted randomly to reduce instrumental and biological bias.

The untargeted analyses were adapted from[1]. An ACQUITY UPLC was used, coupled to a XEVO-G2XS Quadrupole Time-of-Flight (QToF) mass spectrometer (Waters, Manchester, UK) equipped with an ESI (Electrospray Ionization) source, operated both in positive (ESI+) and negative (ESI-) ionization modes. Chromatographic analysis was performed using an ACQUITY UPLC® CSH C18 column (C18, 2.1 mm x 100 mm x 1.7 µm, Waters), employing the mobile phase A composed of water + 0.1% formic acid, and the mobile phase B, composed of Acetonitrile. The flow rate was 0.4 mL min-1. Initially, the column was conditioned with 10% B and maintained for 2 min, increasing to 40% B over the next 0.5 min, increasing to 90% B over the next 4.5 min, and staying for 2 min at this rate. In 2.0 min, mobile phase B returned to 10% and was maintained for another 2.0 min, equilibrating the column for the next injection. The total running time was 13 min. The injection volume was 2 µL. The mass spectrometer was operated in MS^E mode with an m/z range of 50–1200 Da, and an acquisition time of 0.5s per scan. MS^E analysis was operated at 6 V for low collision energy and a ramp of 20-50 V for high collision energy. Leucine enkephalin (molecular weight = 555.62; 200 pg L^{-1} in 1:1 ACN: H2O, v/v) was used as the lock mass for mass accuracy, and a 0.5 mM sodium formate solution was used for calibration. Other parameters were as follows: source temperature = 135 °C, desolvation temperature = 550 °C, desolvation gas flow = 900 L h-1, capillary voltage = 3.2 kV (ESI+) / 3.0 kV (ESI-), and cone voltage = 40 V. Raw data was processed using the Progenesis™ QI v2.4 software (Nonlinear Dynamics, Newcastle, UK), The LC-MS raw files were processed using the Progenesis™ QI software v2.4 (Nonlinear Dynamics, Newcastle, United Kingdom), which allowed the selection of possible adducts, peak alignment, deconvolution, and compound annotation based on MS^E experiments. The adducts [M+H]+, [M+K]+, [M+Na]+, [M+ACN+H]+, [M+H-H2O]+, and [M+NH4]+ were considered for the positive acquisition mode, while [M-H]-, [M+Cl]-, [M-H2O-H]-, [M+FA-H]-, and [M+Na-2H]- were considered for the negative acquisition mode. For each sample, Progenesis QI generates an intensity table of the ions, labeled according to their retention time and nominal masses, called features, as a function of their intensity (areas of the extracted ion chromatogram). The features table obtained from Progenesis QI was then processed using R version 4.2.3 Shortstop Beagle© (The R Foundation for Statistical Computing, 2023). Missing

values were replaced by the minimum value of the data set. Afterward, the relative standard deviation (%RSD) was calculated for the QC samples and only the features with an %RDS < 30% in the QC samples were kept. Data from positive and negative ionization modes were merged in one table for further statistical analysis.

For the targeted analysis, we used a Waters Quattro Micro triple quadrupole mass spectrometer equipped with a Shimadzu SIL-20A LC system, according to the methodology previously[2]. The methodology was performed using FIA without any chromatographic separation, and 10 μL was used as injection volume. The mobile phase was composed of water:acetonitrile:formic acid (80:20:0.1 v/v/v). A flow gradient was used, starting with a zeroed flow until 0.5 min. The zeroed flow was used to allow the integration of the entire peak, with no cuts due to the proximity to the y-axis. Afterward, the flow ranged from 0 to 0.5 mL min-1 from 0.5 to 0.51 min, when it was kept till 3.50 min, then decreasing to 0.1 mL min-1 in a total run-time of 4 min. Multiple reaction monitoring (MRM) transitions were optimized for each compound by analyzing analytical standards described in Supplementary Table 1 (ST1). The acquisition was controlled by the Target Lynx software (Waters). After peak integration, RSD was evaluated for QC samples and only the analytes with RSD<15 % were kept for statistical analysis.

1.2. Statistical Analysis

Data from untargeted and targeted analysis were uploaded separately into the Metaboanalyst 6.0 web platform using a table containing the areas of the extracted ion chromatograms against the sample's names and classes. Data was normalized by the sum, log-transformated, and scaled using Pareto. The selection of the metabolites was based on the One-way ANOVA ($p<0.05$) followed by Tukey's posthoc analysis (p <0.05). PCA was used for data visualization. Heatmaps were built based on ANOVA's selection.

1.3. Biological activity in silico prediction.

All molecules were also individually processed through PASS Online (www.way2drug.com/passonline/) to predict their biological activities with probabilities of activity (Pa) >0.7 and inactivity (Pi) <0.3, where Pa>Pi [3]. Tables S4-S7 show the top 10 of the highest Pa predicted biological activity of each component analyzed here. Table S4 shows that Fucosterol-epoxide lyase inhibitor, Levanase inhibitor, Chymosin inhibitor, Acrocylindropepsin inhibitor, Saccharopepsin inhibitor, Polyporopepsin inhibitor, Procollagen N-endopeptidase inhibitor, Glucan endo-1,3-beta-D-glucosidase inhibitor, and Peptide-N4-(N-acetyl-beta-glucosaminyl) asparagine amidase inhibitor are biological activities related to PEA. Table S5 presents that Feruloyl esterase inhibitor, Beta-carotene 15,15'-monooxygenase inhibitor, Aspulvinone dimethylallyltransferase inhibitor, Membrane integrity agonist, Monophenol monooxygenase inhibitor, Reductant Carminative Linoleate diol synthase inhibitor, and Gluconate 2-dehydrogenase (acceptor) inhibitor are biological activities related to Curcumin. Table S6 presents that Janus Quinase 2 (JAK2) expression inhibitor, HIF-1A expression inhibitor, Preneoplastic conditions treatment, 1-Acylglycerol-3-phosphate O-

acyltransferase inhibitor, Mucositis treatment, Feruloyl esterase inhibitor, Matrix metalloproteinase-9 (MMP9) expression inhibitor, Beta-carotene 15,15'-monooxygenase inhibitor, Monophenol monooxygenase inhibitor, and Tumor necrosis factor (TNF) expression inhibitor are biological activities related to demethoxycurcumin. Table S7 presents that HIF-1A expression inhibitor, JAK2 expression inhibitor, Mucositis treatment, Preneoplastic conditions treatment, Reductant, Membrane integrity agonist, Aspulvinone dimethylallyltransferase inhibitor, Feruloyl esterase inhibitor, Beta-carotene 15,15'-monooxygenase inhibitor, and GST A substrate are biological activities related to bisdemethoxycurcumin.

Supplementary Tables

Table S1: **Amino Acids and Acylcarnitines investigated in *C. elegans* samples, their experimental detection parameters, and the RSD (%) of metabolites for quality control (QC) samples**

Compound	Cone (V)	Collision (eV)	MRM (Transition, *m/z*)	RSD QC (%)
		Amino acids		
Alanine	20	8	90.1-44.1	2.00
Arginine	30	21	175.1-70.1	6.63
Asparagine	20	6	133.1-87.0	2.74
Cysteine	20	10	122.1-105.0	77.12
Citrulline	20	14	176.0-113.0	31.48
Glutamic Acid	20	8	148.1-130.0	4.74
Glutamine	20	8	130.0-84.1	58.30
Glycine	20	7	76.0-30.1	156.93
Histidine	20	16	156.1-110.0	5.27
Leucine	20	9	132.1-85.1	5.88
Lysine	14	14	147.1-84.0	5.10
Methionine	20	9	150.0-104.0	7.01
Ornithine	15	14	133.0-70.0	16.98
Phenylalanine	20	12	166.1-120.1	2.59
Proline	20	10	116.1-70.0	1.78
Serine	14	8	106.1-60.0	3.19
Threonine	38	20	120.1-74.0	8.92
Tryptophan	16	18	205.1-146.0	5.62
Tyrosine	20	12	182.1-136.1	5.52
Valine	20	10	118.1-72.1	8.64
		Acylcarnitines		

Free Carnitine (C0)	40	19	162.0-85.0	7.63
Acetyl-Carnitine (C2)	35	19	204.1-85.0	13.02
Propionyl-Carnitine (C3)	35	19	218.1-85.0	57.58
Butyryl-CarnitineButyryl - Carnitine (C4)	35	19	232.1-85.0	4.35
Isovaleryl-Carnitine (C5)	35	19	246.1-85.0	10.09
Hexanoyl-Carnitine (C6)	35	19	260.0-85.0	7.92
Octanoyl-Carnitine (C8)	40	19	288.1-85.0	8.69
Decanoyl-Carnitine (C10)	40	19	316.0-85.0	12.30
Tetradecanoyl - Carnitine (C14)	40	28	372.0-85.0	5.77
Palmitoyl-Carnitine (C16)	40	25	400.3-85.0	5.90
Hydroxypalmitoyl-Carnitine (C16OH)	40	25	416.0-85.0	4.14
Octadecanoyl-Carnitine (C18)	40	19	428.0-85.0	8.61
Octadecenoyl-Carnitine (C18:1)	40	19	426.0-85.0	3.92
Malonyl-Carnitine (C3DC)	40	19	248.0-85.0	29.01

*Compounds in red indicate metabolites excluded by RSD >15%; MRM: multiple reaction monitoring transitions of mass-to-charge (*m/z*) of precursors-fragment ions

Table S2. The *p*-value for one-way ANOVA followed by Tukey's post hoc analysis ($p \leq 0.05$).

**Compound**	**ANOVA (*p-value*)**	**Post-hoc (UP - DOWN)**
Alanine	0.0005	DTT - S + DTT / S - DTT / Control - S + DTT / S - S + DTT
Arginine	< 0.0001	S + DTT - DTT / Control - DTT / S - DTT / S + DTT - Control / S - Control
Asparagine	0.0384	DTT - S / Control - S

Glutamic Acid	> 0.05	-
Histidine	0.0002	S + DTT - DTT / S - DTT / S + DTT - Control / S - Control
Leucine	0.0001	DTT - S + DTT / DTT - S / Control - S + DTT / Control - S
Lysine	0.0212	S + DTT - DTT / S - DTT / S + DTT - Control / S - Control
Methionine	0.0186	DTT - S + DTT / Control - S + DTT
Phenylalanine	> 0.05	-
Proline	0.0007	S + DTT - DTT / DTT - Control / S + DTT - Control / S + DTT - S / S - Control
Serine	0.0318	DTT - S + DTT / DTT - Control / DTT - S
Threonine	0.0079	DTT - S + DTT / DTT - Control / S - S + DTT
Tryptophan	> 0.05	-
Tyrosine	> 0.05	-
Valine	> 0.05	-
Free Carnitine (C0)	> 0.05	-
Acetyl-Carnitine (C2)	> 0.05	-
Butyryl-CarnitineButyryl -	0.0222	Control - DTT / Control - S + DTT / Control - S
Carnitine (C4)	> 0.05	-
Isovaleryl-Carnitine (C5)	> 0.05	-
Hexanoyl-Carnitine (C6)	> 0.05	-
Octanoyl-Carnitine (C8)	> 0.05	-
Decanoyl-Carnitine (C10)	0.0166	Control - DTT / S + DTT - S / Control - S
Tetradecanoyl - Carnitine	0.0215	DTT - S + DTT / Control - S + DTT / S - S + DTT
(C14)	> 0.05	-
Palmitoyl-Carnitine (C16)	> 0.05	-
Hydroxypalmitoyl-	> 0.05	-
Carnitine (C16OH)		
Octadecanoyl-Carnitine		
(C18)		
Octadecenoyl-Carnitine		
(C18:1)		

DTT: dithiothreitol; S: supplement.

Table S3. Differential Annotated lipids found by one-way ANOVA ($p \leq 0.05$) followed by Tukey's post hoc analysis ($p < 0.05$).

m/z measured	Rt	Adducts	Name	Fórmula	Mass Error (*ppm*)	Post-hoc (UP - DOWN)
496.3392	8,19	M+H	LPC 16:0	C24H50NO7P	-1.07	Control - S / S - S + DTT / Control - S + DTT / DTT - S / Control - DTT / DTT - S + DTT
546.3552	6,64	M+Na	LPC 18:0	C26H54NO7P	4.18	S - Control / S - S + DTT / S + DTT - Control / DTT - Control
520.3390	6,46	M+H	LPC 18:2	C26H50NO7P	-1.46	S - Control / S - S + DTT / DTT - Control / DTT - S + DTT
468.3071	5,95	M+H	LPC 14:0	C22H46NO7P	-2.82	Control - S + DTT / DTT - S + DTT
466.2923	6,62	M+H	LPC 14:1	C22H44NO7P	-0.94	S + DTT - Control / DTT - S / DTT - Control
494.3240	6,04	M+H	LPC 16:1	C24H48NO7P	-0.12	Control - S / S + DTT - S / DTT - S / DTT - Control
522.3555	6,99	M+H	LPC 18:1	C26H52NO7P	0.16	S + DTT - S / S + DTT - Control / DTT - S /DTT - Control / DTT - S + DTT
516.3087	5,71	M+H	LPC 18:4	C26H46NO7P	0.49	S + DTT - Control / DTT - Control
586.3138	5,93	M+FA-H	LPC 20:5	C28H48NO7P	-2.24	Control - S / S - S + DTT / Control - S + DTT / S - DTT /Control - DTT / DTT - S + DTT
464.2761	5,91	M+H-H2O	PC 14:0	C22H44NO8P	-2.11	Control - S / Control - S + DTT / DTT - S
440.2765	6,07	M+H	PC 12:0	C20H42NO7P	-1.38	S - Control / S - S + DTT / S + DTT - Control / DTT - S /DTT - Control / DTT - S + DTT
626.3574	6,34	M+Cl	PC 22:1	C30H58NO8P	-3.37	Control - S / S + DTT - S / DTT - S / DTT - Control / DTT - S + DTT
426.2609	5,76	M+H	LPE 14:0	C19H40NO7P	-1.29	Control - S / S - S + DTT / Control - S + DTT /S - DTT / Control - DTT

452.2766	6,01	M+H	LPE 16:1	C21H42NO7P	-1.05	S - S + DTT / Control - S + DTT / S - DTT / Control - DTT
526.3131	6,27	M+FA-H	LPE 18:0	C23H48NO7P	-3.97	Control - S / Control - DTT / S + DTT - DTT
478.2927	6,80	M-H	LPE 18:1	C23H46NO7P	-2.52	S - S + DTT / Control - S + DTT / S - DTT / Control - DTT
476.2768	6,30	M-H	LPE 18:2	C23H44NO7P	-2.98	Control - S / S + DTT - S / S + DTT - DTT
476.2781	5,95	M+H	LPE 18:3	C23H42NO7P	2.06	S - Control / S - S + DTT / S + DTT - Control / DTT - Control / DTT - S + DTT
490.3291	8,08	M-H2O-H	LPE 20:0	C25H52NO7P	-2.34	Control - S / S + DTT - S / Control - S + DTT / DTT - S / Control - DTT
508.3388	7,84	M+H	LPE 20:1	C25H50NO7P	-1.75	S - S + DTT / Control - S + DTT / S - DTT / Control - DTT
500.2768	6,32	M-H	LPE 20:4	C25H44NO7P	-2.82	S - Control / S - S + DTT
498.2609	5,91	M-H	LPE 20:5	C25H42NO7P	-3.3	Control - S / S - S + DTT / Control - S + DTT / S - DTT / Control - DTT / DTT - S + DTT
580.3608	7,54	M+FA-H	LPE 22:1	C27H54NO7P	-2.16	Control - S / S + DTT - S / DTT - S
450.2608	6,99	M+H-H2O	PE 16:0	C21H42NO8P	-1.32	S - DTT / Control - DTT / S + DTT - DTT
452.2765	6,14	M+H	PE 16:1	C21H42NO7P	-1.32	S + DTT - S / S + DTT - Control / DTT - S / DTT - Control
528.3078	6,04	M+H-H2O	PE 22:3	C27H48NO8P	-1.08	S + DTT - S / S + DTT - Control / DTT - S / DTT - Control
634.3890	6,94	M-H2O-H	PE 30:5	C35H60NO8P	1.88	Control - S / Control - S + DTT / Control - DTT

LPC: Lysophosphatidylcholines, PC: phosphatidylcholines; LPE: lysophosphatidylethanolamine; PE: phosphatidylethanolamine; RT: chromatographic retention time; DTT: dithiothreitol; S: supplement.

Table S4. **PEA's Pass Online predicted biological activity.**

**Pa**	**Pi**	**Biological Activity**
0.946	0.002	Fucosterol-epoxide lyase inhibitor
0.924	0.002	Levanase inhibitor
0.914	0.004	Chymosin inhibitor
0.914	0.004	Acrocylindropepsin inhibitor
0.914	0.004	Saccharopepsin inhibitor
0.899	0.006	Polyporopepsin inhibitor
0.888	0.002	Procollagen N-endopeptidase inhibitor
0.881	0.003	Glucan endo-1,3-beta-D-glucosidase inhibitor
0.879	0.003	Peptide-N4-(N-acetyl-beta-glucosaminyl)asparagine amidase inhibitor
0.871	0.003	Macrophage colony stimulating factor agonist

Table S5. **Curcumin's Pass Online predicted biological activity.**

**Pa**	**Pi**	**Biological Activity**
0.936	0.003	Feruloyl esterase inhibitor
0.898	0.001	Beta-carotene 15,15'-monooxygenase inhibitor
0.900	0.008	Aspulvinone dimethylallyltransferase inhibitor
0.887	0.014	Membrane integrity agonist

0.872	0.003	Monophenol monooxygenase inhibitor
0.864	0.003	Reductant
0.833	0.003	Carminative
0.832	0.005	Linoleate diol synthase inhibitor
0.833	0.010	Gluconate 2-dehydrogenase (acceptor) inhibitor
0.826	0.003	HMOX1 expression enhancer

HMOX1: heme oxygenase 1.

Table S6. Demethoxycurcumin's Pass Online predicted biological activity.

**Pa**	**Pi**	**Biological Activity**
0.978	0.001	JAK2 expression inhibitor
0.974	0.002	HIF1A expression inhibitor
0.950	0.001	Preneoplastic conditions treatment
0.946	0.002	1-Acylglycerol-3-phosphate O-acyltransferase inhibitor
0.937	0.004	Mucositis treatment
0.927	0.003	Feruloyl esterase inhibitor
0.919	0.001	MMP9 expression inhibitor
0.908	0.001	Beta-carotene 15,15'-monooxygenase inhibitor
0.908	0.002	Monophenol monooxygenase inhibitor

0.901	0.002	TNF expression inhibitor

JAK2: Janus Quinase 2; HIF: hypoxia-Inducible Factor; MMP9: matrix metalloproteinase-9; TNF: Tumor necrosis factor.

Table S7. Bisdemethoxycurcumin's Pass Online predicted biological activity.

Pa	Pi	Biological Activity
0.980	0.002	HIF1A expression inhibitor
0.962	0.001	JAK2 expression inhibitor
0.924	0.005	Mucositis treatment
0.912	0.002	Preneoplastic conditions treatment
0.908	0.003	Reductant
0.910	0.009	Membrane integrity agonist
0.899	0.008	Aspulvinone dimethylallyltransferase inhibitor
0.894	0.004	Feruloyl esterase inhibitor
0.884	0.002	Beta-carotene 15,15'-monooxygenase inhibitor
0.879	0.004	GST A substrate

JAK2: Janus Quinase 2; HIF: hypoxia-Inducible Factor.

Table S8. Statistical datas related to development assay.

	ANOVA table	*df*	F (DFn, DFd)	P value
**Figure 1A**	**Interaction**	32	F (32, 210) = 52.94	$p < 0.0001$
	**Estágio**	4	F (4, 210) = 1161	$p < 0.0001$
	**Residual**	210		
	**Interaction**	32	F (32, 210) = 18.53	$p < 0.0001$
**Figure 1B**	**Estágio**	4	F (4, 210) = 562.3	$p < 0.0001$
	**Residual**	210		
	**Interaction**	32	F (32, 210) = 15.11	$p < 0.0001$
**Figure 1C**	**Estágio**	4	F (4, 210) = 126.1	$p < 0.0001$
	**Residual**	210		
	**Interaction**	32	F (32, 210) = 7.993	$p < 0.0001$
**Figure 1D**	**Estágio**	4	F (4, 210) = 31.96	$p < 0.0001$
	**Residual**	210		
**Figure 1E**	**Treatment**	8	F (8, 261) = 42.72	$p < 0.0001$
	**Residual**	261		
**Figure 1F**	**Treatment**	8	F (8, 261) = 24.18	$p < 0.0001$
	**Residual**	261		

Table S9. Statistical datas related to GABAergic neuron development.

	ANOVA table	*df*	F (DFn, DFd)	P value
**Figure 2B**	**Treatment**	8	F (8, 278) = 108.9	$p < 0.0001$
	**Residual**	278		
**Figure 2C**	**Treatment**	8	F (8, 319) = 25.04	$p < 0.0001$
	**Residual**	319		

Table S10. Statistical datas related to stress responses.

	ANOVA table	*df*	F (DFn, DFd)	P value
**Figure 3A**	**Interaction**	12	F (12, 144) = 27.16	$p < 0.0001$
	**Subject**	36	F (36, 144) = 8.04	$p < 0.0001$
	**Residual**	144		
**Figure 3B**	**Interaction**	12	F (12, 144) = 23.22	$p < 0.0001$
	**Subject**	36	F (36, 144) = 9.7	$p < 0.0001$
	**Residual**	144		
**Figure 3C**	**Interaction**	12	F (12, 144) = 18.77	$p < 0.0001$
	**Subject**	36	F (36, 144) = 8.7	$p < 0.0001$

	**Residual**	144		
**Figure 3D**	**Interaction**	12	F (12, 144) = 20.73	$p < 0.0001$
	**Subject**	36	F (36, 144) = 11.53	$p < 0.0001$
	**Residual**	144		
**Figure 3E**	**Interaction**	12	F (12, 112) = 22.18	$p < 0.0001$
	**Subject**	28	F (28, 112) = 6.5	$p < 0.0001$
	**Residual**	112		
**Figure 3F**	**Interaction**	12	F (12, 112) = 19.52	$p < 0.0001$
	**Subject**	28	F (28, 112) = 2.3	$p = 0.0009$
	**Residual**	112		
**Figure 3G**	**Interaction**	12	F (12, 144) = 27.10	$p < 0.0001$
	**Subject**	36	F (36, 144) = 9.7	$p < 0.0001$
	**Residual**	144		

Table S11. Statistical datas related to DAF-16.

	**ANOVA table**	***df***	**F (DFn, DFd)**	**P value**
**Figure 4A**	***Nuclear***			
	**Treatment**	3	F (3, 12) = 4.6	$p = 0.02$
	**Residual**	12		

	ANOVA table	*df*	F (DFn, DFd)	P value
	Intermediate			
	**Treatment**	3	F (3, 12) = 2.7	*p* = 0.08
	**Residual**	12		
	Nuclear			
	**Treatment**	3	F (3, 12) = 1.5	*p* = 0.26
	**Residual**	12		
**Figure 4B**	**ANOVA table**	***df***	**F (DFn, DFd)**	**P value**
	Nuclear			
	**Treatment**	3	F (3, 12) = 4.7	*p* = 0.02
	**Residual**	12		
	Intermediate			
	**Treatment**	3	F (3, 12) = 2.3	*p* = 0.1
	**Residual**	12		
	Nuclear			
	**Treatment**	3	F (3, 12) = 8.8	*p* = 0.002
	**Residual**	12		
	**ANOVA table**	***df***	**F (DFn, DFd)**	**P value**
	Nuclear			
	**Treatment**	3	F (3, 12) = 3.5	*p* = 0.04

	**Residual**	12		
**Figure 4C**	***Intermediate***			
	**Treatment**	3	F (3, 12) = 1.6	*p* = 0.2
	**Residual**	12		
	Nuclear			
	**Treatment**	3	F (3, 12) = 7.4	*p* = 0.004
	**Residual**	12		
	**ANOVA table**	***df***	**F (DFn, DFd)**	**P value**
	Nuclear			
	**Treatment**	3	F (3, 12) = 4.1	*p* = 0.03
	**Residual**	12		
**Figure 4D**	***Intermediate***			
	**Treatment**	3	F (3, 12) = 1.9	*p* = 0.1
	**Residual**	12		
	Nuclear			
	**Treatment**	3	F (3, 12) = 8.7	*p* = 0.002
	**Residual**	12		
	**ANOVA table**	***df***	**F (DFn, DFd)**	**P value**
	Nuclear			

	**Treatment**	3	F (3, 8) = 4.9	*p* = 0.03
	**Residual**	8		
**Figure 4E**	***Intermediate***			
	**Treatment**	3	F (3, 8) = 1.6	*p* = 0.2
	**Residual**	8		
	Nuclear			
	**Treatment**	3	F (3, 8) = 1.6	*p* = 0.1
	**Residual**	8		
	**ANOVA table**	***df***	**F (DFn, DFd)**	**P value**
	Nuclear			
	**Treatment**	3	F (3, 8) = 7.6	*p* = 0.01
**Figure 4F**	**Residual**	8		
	Intermediate			
	**Treatment**	3	F (3, 8) = 2.7	*p* = 0.1
	**Residual**	8		
	Nuclear			
	**Treatment**	3	F (3, 8) = 2.5	*p* = 0.1
	**Residual**	8		
	**ANOVA table**	***df***	**F (DFn, DFd)**	**P value**

Figure 4G	*Nuclear*			
	**Treatment**	3	F (3, 12) = 7.2	*p* = 0.004
	**Residual**	12		
	Intermediate			
	**Treatment**	3	F (3, 12) = 3.7	*p* = 0.04
	**Residual**	12		
	Nuclear			
	**Treatment**	3	F (3, 12) = 8.3	*p* = 0.002
	**Residual**	12		

Table S12. Statistical datas related to SOD-3.

	ANOVA table	*df*	F (DFn, DFd)	P value
**Figure 5A**	**Treatment**	3	F (3, 116) = 24.48	*p* < 0.0001
	**Residual**	116		
**Figure 5B**	**Treatment**	3	F (3, 116) = 7.45	*p* = 0.0001
	**Residual**	116		
**Figure 5C**	**Treatment**	3	F (3, 116) = 5.7	*p* = 0.0010
	**Residual**	116		
**Figure 5D**	**Treatment**	3	F (3, 116) = 10.09	*p* < 0.0001
	**Residual**	116		

**Figure 5E**	**Treatment**	3	F (3, 116) = 8.8	$p < 0.0001$
	**Residual**	116		
**Figure 5F**	**Treatment**	3	F (3, 116) = 9.1	$p < 0.0001$
	**Residual**	116		
**Figure 5G**	**Treatment**	3	F (3, 116) = 7.7	$p < 0.0001$
	**Residual**	116		

Supplementary Figures

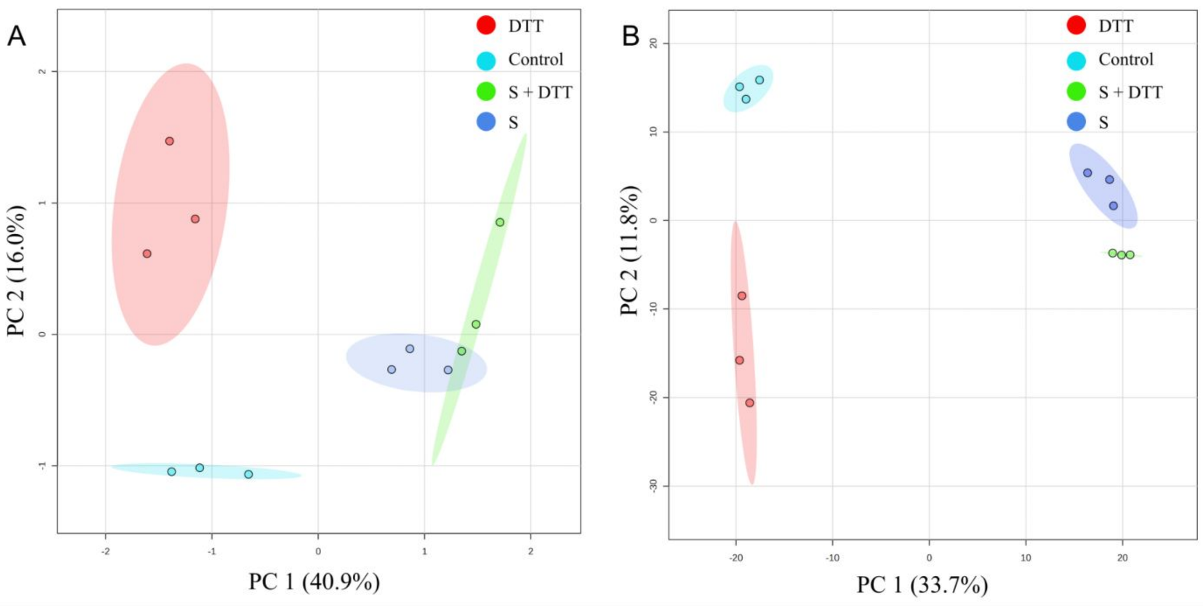

Figure S1. Principal Component Analysis (PCA) results. (A) Results from Targeted analysis. (B) Results form untargeted analysis. The ellipses indicate the confidence interval (CI=95%).

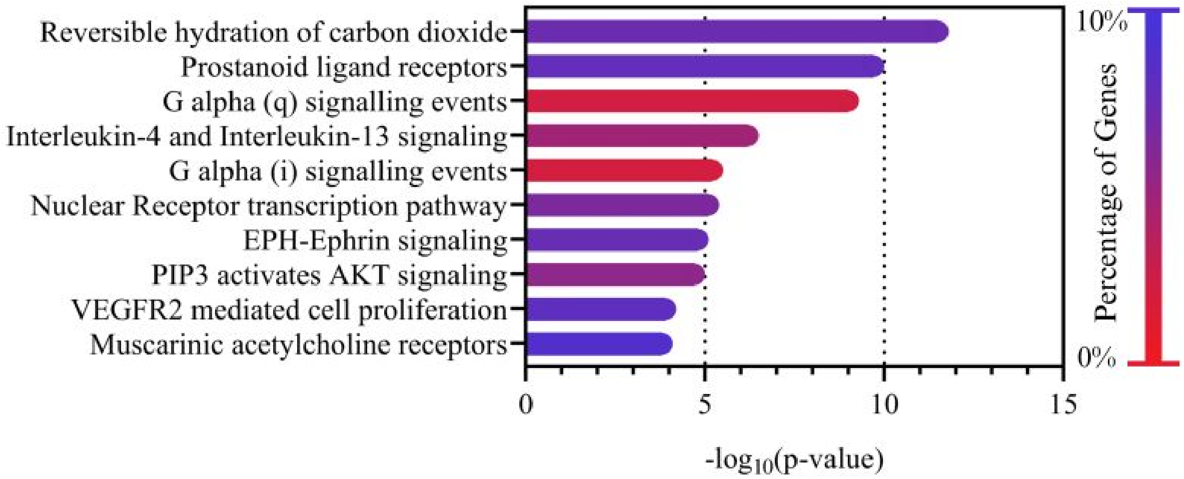

Figure S2. UniProt enrichment analysis of the Reactome pathways through FunRich. EPH: Erythropoietin-producing human hepatocellular; PIP3: Phosphatidylinositol (3,4,5)-trisphosphate; AKT: Protein Kinase B; VEGFR2: Vascular endothelial growth factor receptor 2.

References

(1) Pelosi, A. C.; Fernandes, A. M. A. P.; Maciel, L. F.; Silva, A. A. R.; Mendes, G. C.; Bueno, L. F.; Silva, L. M. F.; Bredariol, R. F.; Santana, M. G.; Porcari, A. M.; Priolli, D. G. Liquid Chromatography Coupled to High-Resolution Mass Spectrometry Metabolomics: A Useful Tool for Investigating Tumor Secretome Based on a Three-Dimensional Co-Culture Model. *PLoS One* 2022, *17* (9), e0274623. https://doi.org/10.1371/journal.pone.0274623.

(2) Moura, A. V.; de Oliveira, D. C.; Silva, A. Ap. R.; da Rosa, J. R.; Garcia, P. H. D.; Sanches, P. H. G.; Garza, K. Y.; Mendes, F. M. M.; Lambert, M.; Gutierrez, J. M.; Granado, N. M.; dos Santos, A. C.; de Lima, I. L.; Negrini, L. D. de O.; Antonio, M. A.; Eberlin, M. N.; Eberlin, L. S.; Porcari, A. M. Urine Metabolites Enable Fast Detection of COVID-19 Using Mass Spectrometry. *Metabolites* 2022, *12* (11), 1056. https://doi.org/10.3390/metabo12111056.

(3) Filimonov, D. A.; Lagunin, A. A.; Gloriozova, T. A.; Rudik, A. V.; Druzhilovskii, D. S.; Pogodin, P. V.; Poroikov, V. V. Prediction of the Biological Activity Spectra of Organic Compounds Using the Pass Online Web Resource. *Chem Heterocycl Compd (N Y)* 2014, *50* (3), 444–457. https://doi.org/10.1007/s10593-014-1496-1.
